# Novel prosthecate bacteria from the candidate phylum Acetothermia

**DOI:** 10.1038/s41396-018-0187-9

**Published:** 2018-06-08

**Authors:** Liping Hao, Simon Jon McIlroy, Rasmus Hansen Kirkegaard, Søren Michael Karst, Warnakulasuriya Eustace Yrosh Fernando, Hüsnü Aslan, Rikke Louise Meyer, Mads Albertsen, Per Halkjær Nielsen, Morten Simonsen Dueholm

**Affiliations:** 1 Department of Chemistry and Bioscience, Center for Microbial Communities, Aalborg University, Aalborg, Denmark; 2 Interdisciplinary Nanoscience Center, Aarhus University, Aarhus, Denmark

## Abstract

Members of the candidate phylum Acetothermia are globally distributed and detected in various habitats. However, little is known about their physiology and ecological importance. In this study, an operational taxonomic unit belonging to Acetothermia was detected at high abundance in four full-scale anaerobic digesters by 16S rRNA gene amplicon sequencing. The first closed genome from this phylum was obtained by differential coverage binning of metagenomes and scaffolding with long nanopore reads. Genome annotation and metabolic reconstruction suggested an anaerobic chemoheterotrophic lifestyle in which the bacterium obtains energy and carbon via fermentation of peptides, amino acids, and simple sugars to acetate, formate, and hydrogen. The morphology was unusual and composed of a central rod-shaped cell with bipolar prosthecae as revealed by fluorescence in situ hybridization combined with confocal laser scanning microscopy, Raman microspectroscopy, and atomic force microscopy. We hypothesize that these prosthecae allow for increased nutrient uptake by greatly expanding the cell surface area, providing a competitive advantage under nutrient-limited conditions.

## Introduction

Microorganisms drive the major biogeochemical nutrient cycles, which are fundamental for many biotechnological processes and directly linked to our health [1–3]. Culture-independent surveys of bacterial communities based on amplicon sequencing of 16S rRNA genes or concatenated single-copy phylogenetic marker genes have revolutionized our understanding of microbial community dynamics and diversity [3–6]. However, such analyses also reveal that many bacterial lineages lack cultivated representatives, and the bacteria affiliated to these candidate lineages are often poorly described [6–8]. These uncharted branches of the tree of life contain valuable information about the evolution of bacteria, exciting novel metabolic pathways, and hitherto unknown functions in microbial communities [6, 9–12].

The fast developments in next-generation sequencing and metagenomics enable the characterization of the whole community gene pool and can be used to elucidate the functional potential of individual microbial members. This allows us to better understand the ecological roles and interactions of the ubiquitous uncultivated microorganisms [13–16]. Genomes of uncultured microorganisms can be recovered from deeply sequenced metagenomes using different methodologies, such as the differential coverage binning approach [14]. Such attempts have been made to establish metabolic models and predict the ecophysiology of several candidate bacteria, such as *Candidatus* Fermentibacter daniensis (candidate phylum Hyd24-12) [17], OP9/JS1 (candidate phylum Atribacteria) [18], and *Candidatus* Promineofilum breve (phylum Chloroflexi) [19].

In one of our studies of anaerobic sludge digesters [17], a metagenome-assembled genome (MAG) classified to the candidate phylum Acetothermia (former OP1) [8] was found to be present in high abundance. The first draft MAG from this phylum was obtained from a subsurface microbial mat in the hot water stream [20]. It was predicted to possess a folate-dependent acetyl-CoA pathway of CO_2_ fixation and have an acetogenic lifestyle. Accordingly, it was given the name *Candidatus* Acetothermum autotrophicum. Another MAG (Acetothermia bacterium 64_32) was extracted from a marine shelf siliciclastic sandstone deposit from an oil reservoir [9]. This draft genome, however, lacked essential genes encoding for autotrophic CO_2_ fixation pathways, indicating a heterotrophic lifestyle. Other physiological information about this candidate phylum is currently not available.

Acetothermia bacteria occupy diverse habitats and have been detected in several geographically separated anaerobic digesters (Figure S1), suggesting that some members of this phylum may be specifically suited for this environmental niche and play a role in the conversion of organic matter into biogas. This motivated us to conduct a detailed investigation into the phylogeny, morphology, physiology, and ecology of Acetothermia bacteria in anaerobic digesters using amplicon sequencing, metagenomics, and advanced visualization techniques. This allows us, for the first time, to reveal an unusual morphology and physiology of this unrecognized microbial player in anaerobic digesters.

## Materials and methods

### Sample collection and storage

Between 1 and 10 biomass samples were obtained from each of 31 anaerobic digesters treating primary and surplus sludge at 18 Danish wastewater treatment plants (WWTPs) in the period from 2011 to 2017 (Table S1, Supplementary Data Set1). A volume of 50 mL digester biomass was sampled, homogenized, and stored as 2 mL aliquots at −80°C before DNA extraction. DNA was extracted using the FastDNA Spin kit for soil (MP Biomedicals, Santa Ana, CA, USA) as optimized for anaerobic digesters by Kirkegaard et al. [17].

### Amplicon sequencing of the 16S rRNA gene

The V4 variable region of the bacterial and archaeal 16S rRNA gene was amplified with the PCR primers 515 F [21] (3′-GTGCCAGCMGCCGCGGTAA-5′) and m806R (3′-GGACTACNVGGGTWTCTAAT-5′) and sequenced using the Illumina platform as described by Albertsen et al. [22]. The m806R primer is a modified version of 806R [21], in which the degeneracy of a single base is increased to ensure a perfect match to all Acetothermia sequences in the SILVA SSURef NR 99 database (Release 128) [23]. The detailed procedures are supplied in Supplementary Methods 1.1.

### Illumina sequencing, metagenome assembly, and genome binning

DNA extracts from four samples collected at different time points during the first half of 2016 from a mesophilic digester at Randers WWTP were used as templates for preparing metagenome libraries and Illumina sequencing (Table S2), as detailed in Supplementary Methods 1.1. The metagenomic assembly and binning process was performed as described by Kirkegaard et al. [17].

### Nanopore sequencing

Genomic DNA was prepared for 1D nanopore sequencing (Oxford Nanopore Technologies, UK), following the manufacturer’s protocol (LSK-108) without the optional DNA shearing and DNA repair steps. The library was loaded on a FLO-MIN106 flow cell and sequenced using the MinION Mk1B DNA sequencer (Oxford Nanopore Technologies). The sequencing software used was MinKNOW v. 1.7.3 (Oxford Nanopore Technologies, UK) with the 48-h sequencing workflow (NC_48 h_Sequencing_Run_FLO_MIN106_SQK-LSK108.py). Sequencing reads were base-called using Albacore v. 1.2.1 (Oxford Nanopore Technologies, UK).

### Genome closing and annotation

The SSPACE-LongRead scaffolder v. 1.1 [24] was used to assemble contigs from the Acetothermia genome bin into a single scaffold based on the long Nanopore reads. Gaps in the draft genome were closed using GapFiller v. 1.11 [25] or by manual read mapping and extension in CLC Genomics Workbench v. 9.5.2. Finally, the closed genome was manually polished to remove SNPs and ensure a high quality assembly (Table S3). Genome annotation was performed in the ‘MicroScope’ annotation pipeline [26, 27] as described by Kirkegaard et al. [17].

### Phylogeny of the 16S rRNA gene and FISH probe design

Phylogenetic analysis and fluorescence in situ hybridization (FISH) probe design were performed using the ARB software package [28] with the SILVA SSURef NR 99 database (Release 128) [23]. All sequences classified to the Acetothermia phylum from the SSURef database were included, except those from the same study that shared ≥ 99% similarity. Potential probes were assessed in silico with the mathFISH software [29]. The Ribosomal Database Project (RDP) PROBE MATCH function [30] was used to identify non-target sequences with indels [31]. Probe validation and optimization were based on generated formamide dissociation curves, as described by Daims et al. [32], and more details are supplied in Supplementary Results 2.1. The final probes are shown in Table S4 and have been deposited in the probeBase database [33].

### FISH and microscopic analysis

Fresh biomass samples, taken from sludge digesters at Randers and Esbjerg WWTPs, were treated with either ethanol or paraformaldehyde (PFA) for the optimal fixation of Gram-positive and Gram-negative bacteria, respectively. FISH was performed on fixed samples as detailed by Daims et al. [32]. The hybridization conditions applied for each probe are given in Table S4. More detailed procedures are supplied in Supplementary Method 1.3.

Cells hybridized with newly designed probes were observed by a white light laser confocal microscope (Leica TCS SP8 X). Targeted cells were further characterized by Raman microspectrometry using a Horiba LabRam HR 800 Evolution system (Jobin Yvon, France) equipped with a Torus MPC 3000 (UK) solid-state semiconductor laser, and higher resolution information on the cell shape was further obtained by atomic force microscopy (AFM) from Syto9-stained cells, using a JPK Nanowizard IV system (Berlin, Germany) on an inverted Zeiss Axiovert 200 M epifluorescence microscope. The detailed procedures and equipment information are supplied in Supplementary Method 1.4.

### Data availability

The raw amplicon sequencing reads, metagenome reads, and the annotated genome sequence data have been submitted to the European Nucleotide Archive (ENA) under the study accession number PRJEB22104.

## Results and discussion

Acetothermia bacteria have previously been observed in anaerobic digesters [34], but their distribution and abundance in these systems are not known. It was therefore decided to survey the microbial composition of 31 full-scale anaerobic digesters using 16S rRNA gene amplicon sequencing. PCR primers were selected and modified to realize an optimized coverage of Acetothermia in the whole community, as detailed in Supplementary Results 2.2 and Figure S2.

**Fig. 1:**
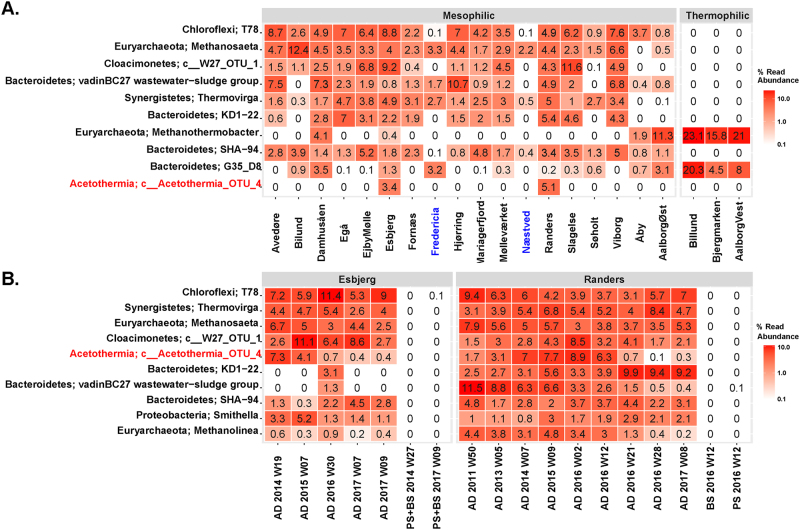
Heatmap of the 10 most abundant microbial genera in anaerobic digesters treating sewage sludge. **a** Average genera abundances of the period of 2011 ~ 2016 in the digesters from 20 wastewater treatment plants (WWTPs). Labels at the bottom of the heatmap represent the location of WWTPs and digesters. Blue labels represent WWTPs applying thermal-hydrolysis process for pre-treating the feedstock. **b** Temporal analysis of the microbiome composition in the digesters from Randers and Esbjerg WWTPs and of the feedstock. Mean abundances of two digesters running in parallel at each WWTP were shown in the profile. Labels at the bottom of the heatmap represent sample type, year, and week of sampling time. Sample type includes: AD for sludge from anaerobic digesters; PS for sludge from the primary clarifier, and BS for surplus biological sludge from secondary clarifier; BS + PS for a mixture of PS and BS before being fed into the digester. Classification levels presented are phylum and genus, which are separated by a semicolon. The genera are sorted by the mean abundance across all the analyzed samples. “(Color figure online)”

Only a single operational taxonomic unit (OTU) assigned to phylum Acetothermia was observed in four mesophilic sludge digesters at two WWTPs from the survey of 31 digesters (Fig. 1a). The two individual WWTPs have no link between each other in terms of operation, seeding microbiome and feedstock, indicating low diversity of Acetothermia bacteria in anaerobic digester ecosystem. The OTU was stably present over a period of 3–6 years in these digesters, but displayed a notable decline from the summer of 2016. It ranked among the five most abundant bacterial OTUs and constituted from 0.1 to 8.9% of all sequenced 16S rRNA gene amplicons (Fig. 1b). The Acetothermia OTU was not detected in amplicons of the incoming feed streams (primary and surplus biological sludge from the wastewater treatment processes), which indicates that the abundance observed was due to growth in the digesters and not immigration. No OTUs related to Acetothermia were observed in thermophilic digesters, or the mesophilic digester operated with thermal hydrolysis of feedstock (Fig. 1a). This indicates that the Acetothermia represented by the OTU has special habitat requirements specific to some mesophilic systems that treat primary and surplus biological sludge.

### Complete genome of the Acetothermia bacterium

To learn more about the ecophysiology of Acetothermia bacteria in anaerobic digesters, we sought to obtain genomic information from the organism represented by the abundant OTU. This organism was consistently found in high abundance in a full-scale anaerobic sludge digester at Randers WWTP, thus providing a good target-system for in-depth investigations (Fig. 1b). To this end, metagenomes were constructed from four individual biomass samples collected during the first half of 2016 (Table S2) and a 12-contig draft genome of Acetothermia bacterium sp. Ran1 (‘Ran1’ in short) was successfully binned from these using differential coverage binning [14] (Figure S3). Longread Nanopore data were obtained from one of the four samples and used to scaffold the draft genome and create a complete closed genome after manual polishing. The characteristics of this genome are shown in Table S3.

### Phylogenetic analyses of Ran1

**Fig. 2:**
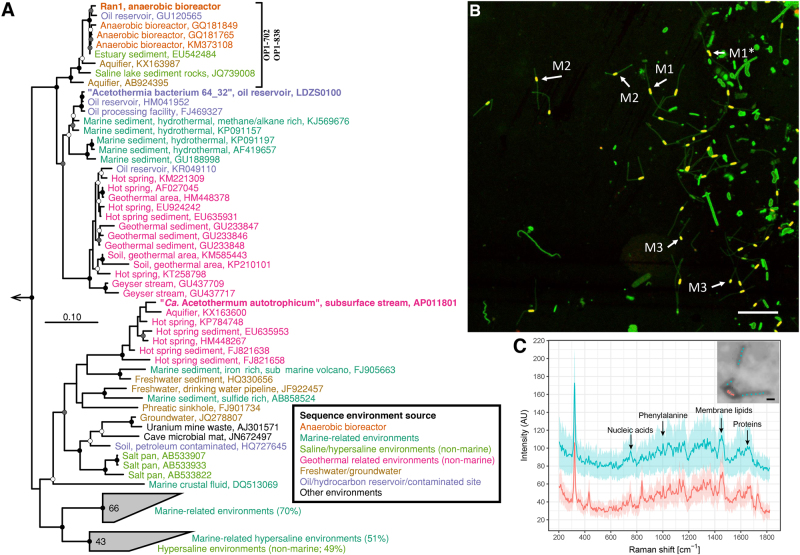
**a** Maximum-likelihood (PhyML) 16S rRNA gene phylogenetic tree of sequences classified to the candidate phylum Acetothermia (SILVA SSURef NR 99, Release 128). The alignment used for the tree applied a 20% conservational filter to remove hypervariable positions, giving 1120 aligned positions. Sequences are colored according to their isolation source environment. Proposed phylogenetic classification of the novel genus and coverage of the newly designed FISH probes are indicated with a black bracket. Bootstrap values from 100 re-samplings are shown for branches with  50 ~ 70% (white dot), 70 ~ 90% (gray) and >90% (black) support. Species of the phylum Thermotogae were used as the outgroup. The scale bar represents substitutions per nucleotide base. An expanded version of the tree is provided in Figure S4. **b** Composite fluorescence micrograph of the Acetothermia cells, hybridized with the OP1-702 FISH probe (Cy3, red), and stained with Syto9 (green). Yellow signal indicates overlap of fluorescence from Cy3 and Syto9. Arrows indicate three slightly different morphologies: M1 = central rod with bipolar prosthecae of similar length; M2 = smaller central rod with bipolar prosthecae of different lengths; M3 = smallest central rod with a single polar prostheca. An M1 cell which seems to be undergoing cell division is indicated with an asterisk. Scale bar represents 10 μm. **c** Raman spectra of the prosthecae and main body of a bipolar prosthecate cell targeted by OP1-702 probe. Seven spectra for the main rod body (red) and 13 for the prosthecae (cyan) were obtained from different sections of the cell as indicated, respectively, by the red and cyan spots on the embedded cell image. Average spectra of the rod (red) and prosthecae (cyan) are shown with the standard deviation depicted as ribbons. Peaks assigned for nucleic acids (784 cm^−1^), phenylalanine (1004 cm^−1^), membrane lipids (1450 cm^−1^), and amide I linkages of proteins (1660 cm^−1^) [76, 77] are indicated by black arrows. PFA-fixed biomass samples were used for the observations in **b**, **c**, originating from an anaerobic sludge digester at Randers WWTP. “(Color figure online)”

**Fig. 3:**
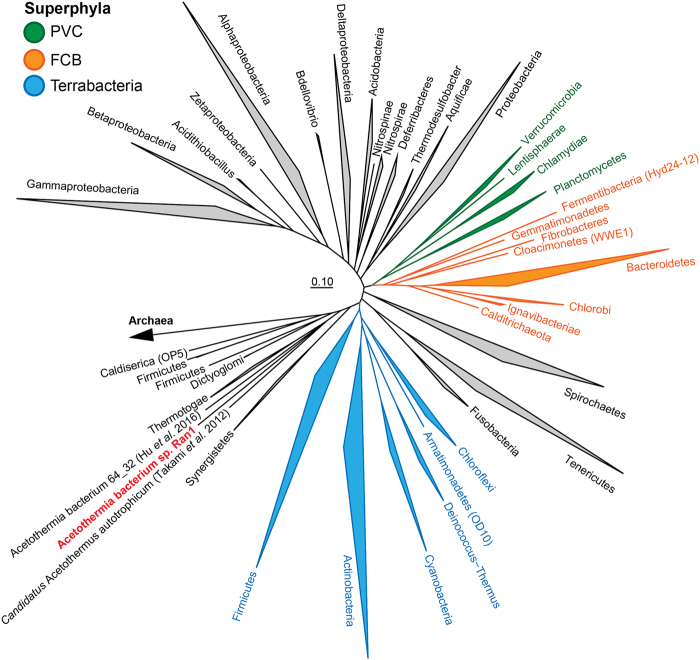
Phylogenetic position of Acetothermia genomes in the reference genome tree generated by CheckM v. 1.0.6 [78] and visualized in ARB v. 6.0.2 [28]. The CheckM tree is inferred from the concatenation of 43 conserved marker genes and incorporates 2052 finished and 3605 draft genomes from the IMG database [78]. “(Color figure online)”

Ran1 was classified to the Acetothermia phylum based on its 16S rRNA gene using the SILVA taxonomy. Phylogenetic analyses of the available sequences for this phylum revealed evident separation of lineages with similar ecological preferences and habitats (Fig. 2a, Figure S4). The 16S rRNA gene sequence of Ran1 clustered into a mono-phylogenetic group together with sequences from other anaerobic digesters [34–38]. Based on the recommended sequence similarity cutoff values for the definition of phylogenetic taxa [39], this group represents a new genus, within the same family as the uncultured Acetothermia bacterium 64_32 [9]. A phylogenetic tree based on concatenated single-copy marker genes was created and used to establish a broader phylogenetic context (Fig. 3). This revealed that Ran1 and the previous Acetothermia draft genomes [9, 20] are distantly related to all currently available genomes, supporting its status as a novel phylum.

### Morphology

To investigate the morphology of Ran1, we designed two FISH probes that cover the proposed novel genus that contains Acetothermia bacteria associated with anaerobic digesters (Fig. 2a). These probes were then applied to samples from one of the digesters at Randers WWTP (Fig. 2b, S5, and S6). Both PFA- and ethanol-fixed samples were analyzed to ensure optimal fixation of Gram-positive and Gram-negative bacteria, respectively (Figure S6). FISH results revealed single rod-shaped cells (~ 0.8 × 1 ~ 2 μm) dispersed in the liquid phase, which were hybridized with the genus-specific probes, indicating a planktonic lifestyle. With ethanol-fixed biomass, appendages (~ 0.4 × 4 ~ 8 μm) were observed at both poles of the rod-shaped cell. FISH signals for these structures were patchy, indicating a relatively low number of ribosomes present inside the appendages (Figure S6). No FISH signal was observed for the appendages with PFA-fixed cells (see more details in Supplementary Results 2.1.2). When using Syto9 to stain the nucleic acids, these appendages were clearly visualized for the probe-hybridized cells in both PFA- and ethanol-fixed samples (Figure S6). It suggests that the nucleic acid containing cytoplasm was shared between the rod-shaped “main body” and the appendages. This was further confirmed by Raman microspectroscopy analysis, which demonstrated a similar composition of the main body and the appendages in terms of nucleic acids, membrane lipids, and proteins (Fig. 2c). Probe-targeted cells from another digester at Esbjerg WWTP demonstrated similar morphology. Accordingly, we hypothesize that the appendages are extensions of the cell envelope out of the central rod body, similar to the prosthecae of *Caulobacter* and *Asticcacaulis* [40]. Such appendages have previously only been described for bacteria within the class Alphaproteobacteria [41].

Further analysis of FISH data demonstrated three different morphologies according to the size of the central rod and the length or appearance of the polar prosthecae: (1) central rod with bipolar prosthecae of similar length; (2) smaller central rod with bipolar stalks of different length; (3) smallest central rod with a single polar prostheca (Fig. 2b). These different morphologies likely represent sequential development of bacterial morphology at different growth stages, in which small rods with a single prostheca represent cells just after cell division, and the longer rods with two prosthecae of equal length represent cells just before cell division. Indeed, it was possible to identify a few dividing cells with prosthecae of equal length (Fig. 2b). Dynamic morphology changes in a cell cycle is already known from other prosthecate bacteria, such as *Caulobacter* [42] and *Asticcacaulis* [43].

**Fig. 4:**
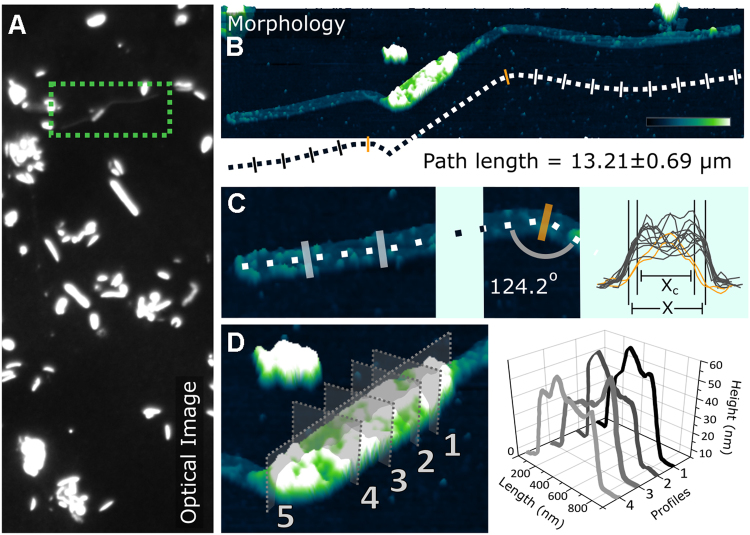
Combined optical and atomic force microscopy images reveal the morphology of one of the Ran1 cells. **a** The optical image to the left shows a broad overview of the sample, which is composed of bacteria of different shapes; **b** The morphology image presents the 3D form of a Ran1 cell in real space. The scale bar is 2 µm in length, and the color transition represents the height change from 0 to 39 nm. **c** The cell stretches out to 13.21 ± 0.6 µm with prosthecae at both poles, which are 0.26053 ± 0.00911 µm (X) in width, except for slight narrowing down to 0.22465 ± 0.00115 µm (X_C_) owing to bending with angles of up to 124.2 ± 3.6°; **d** Zoom in the image of the main rod body. Cross sections show a rugged surface, as depicted and measured by Profile 1–4 perpendicular to the length of the rod represented by Profile 5. “(Color figure online)”

Higher resolution information on cell surface properties of Ran1 was obtained using AFM (Fig. 4). AFM confirmed the morphology observed by FISH microscopy, i.e., a central rod-shaped cell with prosthecae extending from both poles. Analysis of four individual Ran1 cells revealed that the average width and length of the main rod body were 0.46 ± 0.03 µm and 1.58 ± 0.39 µm, respectively. The average height of only 0.066 ± 0.017 µm showed that cells collapsed during air drying of the sample. The width of the prosthecae was relatively constant (0.256 ± 0.004 µm), but decreased to 0.225 ± 0.001 µm in cross sections where bending of the prosthecae occured. Such bendings were observed in most samples, and the degree of narrowing varied, based on the bending angle, which was up to 124.2 ± 3.6°. This indicated flexibility of the prosthecae. The total length of the bacteria with prosthecae was 11.42 ± 1.49 µm.

The total surface area (SA) and surface area to volume ratio (SA/V) were calculated for the rod-shaped cell with and without the prosthecae, based on the observed average length and width. Results show that development of the prosthecae made the SA increase by 3.5 times (from 2.28 to 10.20 µm^2^) and SA/V become 42% larger (from 9.6 to 13.7 μm^−1^), providing an increased interface for nutrients uptake [44–46]. It has been demonstrated that prosthecate bacteria have a competitive advantage under nutrient-deficient conditions, and they are often observed under such conditions [42–44, 46]. Nevertheless, this effect is even more pronounced in diffusion-limited environments, where the rate of nutrient uptake is proportional to the effective linear dimension of a structure, rather than to its SA [46]. Indeed, the length of the prostheca of *Caulobacter* inversely correlates with the availability of phosphate, indicating enhanced phosphate uptake capability [47]. Consistent with this observation, the digesters that harbor Ran1 in abundance demonstrated relatively low soluble orthophosphate concentration (~ 25 ~ 80 mg PO_4_−P/L), compared with the other digesters (95 ~ 480 mg PO_4_−P/L) (Table S1). Furthermore, it was observed that the decrease of Ran1 (from 6 ~ 8% to < 1%) in the summer of 2016 followed an increase of phosphorus content (PO_4_−P and Total P) as well as concentration of organic compounds (VFAs) in the liquid phase (Figure S7 and S8). This supports the idea that Ran1 may have a competitive advantage in nutrient-limited engineered systems, especially reactors with relatively low amounts of phosphorus. Ran1 may, therefore, be used as a bioindicator for such a condition, but more studies are needed to verify this hypothesis.

### Genome inferred surface properties

Some cell envelope properties can be inferred directly from genomes, based on the presence or absence of cell envelope genes found specifically in archetypical monoderm or diderm lineages [14], which are characterized by having one or two cellular membranes, respectively [48]. This study revealed an unusual cell envelope architecture of Ran1, with similarities to both members of the monoderm Chloroflexi and the atypical diderms Thermotoga and Deinococcus-Thermus (Figure S9). Accordingly, it is less than easy to conclude whether Ran1 has a mono- or diderm cell envelope. The genome did not contain any genes associated with lipopolysaccharides, which are commonly found in the outer membrane of diderm bacteria [48]. However, genes encoding an outer membrane-specific bacterial surface antigen and an outer membrane permease imply that Ran1 may have a simple diderm cell envelope similar to those found in Thermotoga [49]. The sheath-like outer membrane of Thermotoga changes its size according to environmental conditions, which has been proposed to provide increased access to nutrients in the same manner as the prosthecae of prothecate bacteria [50]. Accordingly, it may be proposed that the outer membrane of Ran1 is a simple scaffold for high-affinity nutrient transporter [46].

Further genome annotation and specialized searches using the PilFind program [51] did not reveal any genes associated to flagella, fimbriae, pili, or cell surface adhesins. However, a few genes related specifically to prostheca development were encoded by the genome, such as the bactofilin family cytoskeletal protein CcmA and the bifunctional penicillin-binding protein Pbp [42]. In *Caulobacter crescentus*, bactofilins are found as membrane-associated clusters at the pole of the cell, where they recruit the peptidoglycan synthase PbpC and initiate prosthecae development [42]. It is, therefore, likely that Ran1 may use a similar strategy for this purpose.

### Metabolic model for Ran1

**Fig. 5:**
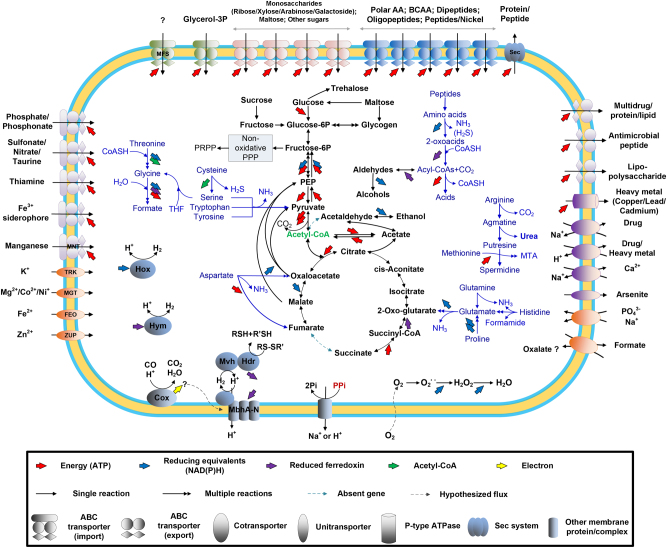
Metabolic model of Acetothermia sp. Ran1 based on the annotated genome sequences (Supplementary Data Set2). AA = Amino acids; BCAA = Branched-chain amino acids; Sec = Secretion system; Glycerol-3P = Glycerol-3-phosphate; PPP = Pentose phosphate pathway; PRPP = 5-Phospho-alpha-D-ribose-1-diphosphate; ATP = Adenosine triphosphate; CoA = Coenzyme A; THF = Tetrahydrofolate; NAD(P)H = Nicotinamide adenine dinucleotide (phosphate) hydrogen; Pi = Phosphate; PPi = Pyrophosphate; MTA = 5’-S-Methyl-5’-thioadenosine; MNT = Manganese transporter; TRK = Potassium (K) transporter; MGT = Magnesium transporter; FED = Ferrous iron (Fe^2+^) transporter; ZUP = Zinc (Zn) transporter; MFS = Major facilitator superfamily transporter. More details on amino acids and electron transport metabolisms are shown in Figure S10–12. “(Color figure online)”

To learn more about the potential function of Ran1, we constructed a metabolic model based on the annotated genome (Fig. 5 and Supplementary Data Set2). A brief overview of the metabolic model is provided below, and detailed descriptions of selected pathways are given in Supplementary Results 2.3.

### Carbon uptake and central metabolism

Several ABC transporter genes were detected, including those for importing amino acids, peptides, glycerol-3-phosphate, maltose, ribose, and alpha-glucoside. This indicates that Ran1 can take up these compounds at the expense of ATP or the proton motive force (PMF) and use them as carbon and energy sources.

Sugars imported can be catabolized through the Embden–Meyerhof–Parnas pathway. The ATP produced during the transformation of hexoses to pyruvate can provide the cells with energy. Besides hexoses, Ran1 may utilize a broad range of pentoses, as it has all the genes of the non-oxidative pentose phosphate pathway [52]. Ran1 also encoded the complete pathway for glycogen metabolism and the gene encoding a trehalose synthase. Therefore, glycogen and trehalose may serve as carbon and energy storage, which can be utilized to mitigate fluctuations in substrate availability [53, 54]. Two extracellular glycosylases were identified, including a cellulase and a glycoside hydrolase. This indicates that Ran1 has some limited extracellular saccharolytic activity and can hydrolyze polysaccharides from the feeding sludge into simpler sugars.

The pyruvate generated from sugars can be converted to acetyl-CoA by the pyruvate:ferredoxin oxidoreductase complex (*porABC*), generating reduced ferredoxin (Fd_red_). Acetyl-CoA can then enter the fermentation pathway catalyzed by two acetyl-CoA synthetases (*acsA* or *acdA*), resulting in the production of acetate and energy in the form of ATP.

The genome encoded an incomplete tricarboxylic acid pathway, in which a succinate dehydrogenase/fumarate reductase complex was not annotated. Considering that no complete electron transport chain for aerobic or anaerobic respiration was found for Ran1, the partial pathway may serve as a source of biosynthetic precursors for anabolic pathways, as in methanogens and some other anaerobic bacteria [55, 56].

Amino acids and peptides, imported by ABC transporters, represent a potential source of carbon, nitrogen, energy, and building blocks of the cell. Indeed, it was found that the genome encoded genes for catabolizing at least 13 of the 22 amino acids (Figure S10 and S11). Serine, glycine, cysteine, aspartate, glutamate, glutamine, histidine, tyrosine, and tryptophan can be deaminated and converted into either pyruvate, oxaloacetate, or 2-oxoglutarate (Figure S10). These intermediates are then further oxidized by the pyruvate:ferredoxin oxidoreductase (*por*) or 2-ketoglutarate ferredoxin oxidoreductase (*kor*) to generate acetyl-CoA or succinyl-CoA, which can then be cleaved to yield acetate or succinate and energy in the form of ATP [57, 58]. Glycine and serine can alternatively be degraded to formate through the glycine cleavage system and tetrahydrofolate pathway [34], concomitant with the generation of ATP and reducing equivalents (in the form of NADH and Fd_red_). Some key enzymes involved in the catabolism of branched-chain amino acids were absent in the annotated genome (Figure S11). It is therefore only the non-branched-chain amino acids that can be used as energy source.

Limited capacity for amino-acid synthesis was encoded in the genome (Supplementary Data Set2), indicating that some of the imported amino acids need to be directly used in anabolic pathways [59].

Ran1 does not have the necessary genes for nitrogen fixation and ammonia import. Amino acids are thus predicted to be a major source of nitrogen, as NH_3_ is produced from deamination and assimilated via the glutamine/glutamate synthase pathway [60]. The high dependence of exogenous amino acids and the fact that Ran1 only encode a single extracellular protease imply its high dependence on the proteolytic action of other members of the microbial community, such as *Thermovirga*, which coexists at high abundance (Fig. 1b) and can hydrolyze proteinous substrates [3, 61].

### Energy conservation and electron flow

Ran1 encodes an energy-conserving, membrane-bounded hydrogenase complex (Mbh A-N) (Fig. 5 and S12), which can translocate protons across the membrane while catalysing Fd_red_-driven H_2_ production [18, 62]. It enables the cell to establish a PMF from Fd_red_ [63]. The produced H_2_ and Fd_ox_ can be recycled by another complex formed by the electron-bifurcating heterodisulfide reductase (Hdr A-C) and the methyl viologen reducing hydrogenase (Mvh D,G,A) [62]. In addition, a bidirectional [NiFe] hydrogenase complex (Hox E,F,U,H,Y) and a putative [Fe] hydrogenase (Hym AB) were also encoded. These complexes catalyse the electron transfer between H^+^/H_2_ with NAD(P)H/NAD(P)^+^ [64, 65] and Fd_red_/Fd_ox_ [66], respectively. These bidirectional hydrogenases are hypothesized to function as electron valves, balancing reductants in the cell [65]. As part of the energy-recycling system, the membrane-integral pyrophosphatase can also translocate H^+^ or Na^+^ to generate PMF, using the energy produced from hydrolysis of pyrophosphate [67]. The H_2_ and acid products (formate and acetate) generated from fermentation can be utilized by the hydrogenotrophic *Methanolinea* and acetotrophic *Methanosaeta* in the system (Fig. 1b).

Surprisingly, the genome does not encode any conventional ATP synthases, which are often used to generate ATP at the expense of the established PMF [68]. Loss of functional ATP synthase has also been reported for other strictly anaerobic fermenters, such as *Clostridium acetobutylicum* [69] and *Clostridium perfringens* [70]. The energy stored in the PMF is therefore most likely used for active transport of substrates [71].

### Stress response

The genome possesses several genes typical of anaerobic bacteria, such as the oxygen-sensitive class III ribonucleoside triphosphate reductase, ferredoxin oxidoreductases, and radical S-adenosyl-methionine-dependent proteins (Supplementary Data Set 2). The oxygen-required class-I and oxygen-tolerant class-II ribonucleotide triphosphate reductases were not found. However, Ran1 encodes several proteins predicted to counter oxidative damage, including superoxide reductase, ruberythrin, thioredoxins, peroxidases, thioredoxin reductase, and glutaredoxins, which may allow it to survive under microaerobic conditions.

### Ecological significance

The heterotrophic way of life predicted for Ran1 is similar to that of the oil reservoir-associated Acetothermia bacterium 64_32 [9], which affiliates to the same family level clade (Fig. 2a). In contrast, another more distantly related member of the phylum, *Candidatus* Acetothermum autotrophicum, is predicted to use chemolithoautotrophic acetogenesis as the primary energy and carbon source, which might reflect the organics-depleted state of its habitat, the microbial mat in hydrothermal ecosystem [20]. Ran1, as a representative member of the anaerobic digester-specific genus, lives a planktonic life in the continuesly-stirred liquid phase (Figs. 2b and 4c). Accordingly, it has direct access to the nutrients introduced from the feedstocks or released from the hydrolytic activities of exoenzymes. The increased surface area due to the prosthecae structure and relatively high surface area to volume ratio compared with ordinary rods, might enable it to compete for substrates with other bacteria in the system. It may play an important role in the anaerobic food web as a consumer of soluble intermediate products (like amino acids) generated by hydrolytic bacteria (like the proteolytic *Thermovirga*), and as a provider of precursor substances (such as acetate, formate, and hydrogen) for methanogens (like the acetotrophic *Methanosaeta* and hydrogenotrophic *Methanolinea*) which coexisted in the ecosystem at high abundance (Fig. 1b).

## Concluding remarks

This study presents the first detailed insight into the morphology, physiology, and ecology of a member of the candidate phylum Acetothermia (former OP1). The bacterium was stably present in several mesophilic sludge digesters during a period of several years and represents a novel genus that includes other previously detected 16S rRNA gene sequences of Acetothermia in anaerobic bioreactors. The metabolic reconstruction suggested that it is an anaerobic, fermentative bacterium involved in acidogenesis, producing organic acids (such as acetate and formate) and hydrogen from the fermentation of peptides, amino acids, and simple sugars (maltose, sucrose). Interestingly, this Acetothermia bacterium demonstrated an unusual morphology composed of a central rod cell and long prosthecae protruding from both poles of the rod. It is the first time this type of morphology has been shown for a bacterium outside the class Alphaproteobacteria, which can shed new light on the evolution of cell morphology. The long and flexible prosthecae greatly expand the surface area of the cell and provide increased access to nutrients under nutrient-limiting conditions. This is supported by their abundance being restricted to digesters with relatively low levels of phosphorus and other nutrients. The genome generated in this study is one of the few closed genomes for uncultured candidate phyla and importantly provides the foundation for future study on pathway expression of the lineage with metatranscriptomics and metaproteomics. The design of FISH probes for the genus will facilitate future in situ studies of the genus in other systems.

### Taxonomic proposals

Phylogenetic analyses of the Ran1 genome classified it as a novel genus within the previously described Acetothermia phylum. We suggest that the closed genome should serve as the type material for this genus [72, 73] and propose the following taxonomic names for the novel genus and species:

*Candidatus* Bipolaricaulis gen. nov.

*Bipolaricaulis* (Bi.po.la.ri.cau’lis. L. adv. num. *bis* twice; N.L. adj. *polaris* polar, pertaining to the poles of the rod-shaped cell; L. masc. n. *caulis* a stalk; N.L. masc. n. *Bipolaricaulis* stalks at both poles).

*Candidatus* Bipolaricaulis anaerobius sp. nov.

*Bipolaricaulis anaerobius* (an.a.e.ro’bi.us. Gr. pref. *an* not; Gr. n. *aer aeros* air; N.L. masc. n. *bius* from Gr. masc. n. *bios* life; N.L. masc. adj. *anaerobius* not living in air, anaerobic).

In addition, we would like to propose that the encompassing Acetothermia phylum be renamed. The phylum takes its name from the type genus *“Candidatus* Acetothermus” [20]. However, *Acetothermus* was already the name of a valid genus within the phylum Bacteroidetes [74, 75], making its reuse illegitimate. The authors also use “*Candidatus* Acetothermum” and “*Candidatus* Acetothermus” interchangeably within the original publication [20]. To prevent confusion, we propose that *Candidatus* Bipolaricaulis anaerobius instead be made the type genus for the phylum and the name be changed accordingly to *Candidatus* Bipolaricaulota (the phylum of the genus *Candidatus* Bipolaricaulis).

## Electronic supplementary material

Supplementary Information

Data Set1

Data Set2

Data Set3
