# Supplementary material for: Novel prosthecate bacteria from the candidate phylum Acetothermia
Source: ISME J. 2018 Jun 8;12(9):2225–37. doi: 10.1038/s41396-018-0187-9 (PMC6092417; doi:10.1038/s41396-018-0187-9)
Supplement: Supplementary file 1 — Supplementary Information [file 41396_2018_187_MOESM1_ESM.docx]

**Supplementary information**

Novel prosthecate bacteria from the candidate phylum Acetothermia

**Authors:** Liping Hao^1^, Simon Jon McIlroy^1^, Rasmus Hansen Kirkegaard^1^, Søren Michael Karst^1^, Warnakulasuriya Eustace Yrosh Fernando^1^, Hüsnü Aslan^2^, Rikke Louise Meyer^2^, Mads Albertsen^1^, Per Halkjær Nielsen^1*^, Morten Simonsen Dueholm^1*^

**Affiliation:**

^1^Center for Microbial Communities, Department of Chemistry and Bioscience, Aalborg University, Aalborg, Denmark.

^2^Interdisciplinary Nanoscience Center, Aarhus University, Aarhus, Denmark

*Correspondence to: Morten Simonsen Dueholm or Per Halkjær Nielsen, Center for Microbial Communities, Department of Chemistry and Bioscience, Aalborg University, Fredrik Bajers Vej 7H, 9220 Aalborg, Denmark; Phone: (+45) 9940 8503; Fax: Not available; E-mail: [md@bio.aau.dk](mailto:md@bio.aau.dk) or phn@bio.aau.dk

# **Supplementary methods**

**1.1 Amplicon sequencing of the 16S rRNA gene**

Amplicon sequencing libraries (**Supplementary Data Set1**) were pooled in equimolar concentrations with a final loading concentration of 10 pM and sequenced on the MiSeq (Illumina) platform using a MiSeq reagent kit v3 (2 × 300 bp).

All sequenced sample libraries were trimmed, and low quality reads were removed using trimmomatic v. 0.32 (Bolger *et al.*, 2014) and then merged using FLASH v. 1.2.11 (Magoč and Salzberg, 2011). The reads were screened for potential PhiX contamination using USEARCH v. 7.0.1090 (Edgar, 2010). The reads were clustered into operational taxonomic units (OTUs, sequence identity ≥ 97%) using USEARCH v. 7.0.1090-cluster_otus with default settings, and subsequently classified using the RDP classifier (Wang *et al.*, 2007) with the MiDAS database v. 1.23 (McIlroy *et al.*, 2017). Further analyses were performed in R environment v. 3.4.1 (R Core Team, 2016) using the R CRAN packages ampvis v. 1.24 (Albertsen *et al.*, 2015) and ggplot2 v. 1.0.1(Wickham, 2009). The samples were subsampled to an even depth of 10,000 reads per sample.

**1.2** **Illumina sequencing, metagenome assembly, and genome binning**

Illumina Nextera DNA Library Prep kit was used to prepare metagenome libraries following the standard protocol. The libraries were paired-end (2 x 250 bp) sequenced on the Illumina HiSeq 2500 platform using the HiSeq Rapid PE cluster kit v2 and the HiSeq Rapid SBS kit v2 (500 cycles) in rapid run mode and also paired-end sequenced (2 x 300 bp) on Illumina MiSeq platform using MiSeq reagent v3 (600 cycles). Standard protocols were used for sample preparation and sequencing.

**1.3 Sample fixation and** **Fluorescence in situ hybridization (FISH)**

For PFA fixation, diluted samples [1:4 in 1 x Phosphate-Buffered Saline (PBS) solution] were first fixed with 4% (w/v) PFA and then stored in 50% (v/v) ethanol / 1 x PBS solution at −20°C, as previously described (Daims *et al.*, 2005). For ethanol fixation, pellets were first obtained by removing the supernatant by centrifugation at 12,000 x g for 5 min at 4°C, and then directly fixed and stored in 50% (v/v) ethanol / 1 x PBS solution.

When FISH was performed on the fixed samples, the NON-EUB probe was applied as a negative control for hybridization (Wallner *et al.*, 1993). Nucleic acids in cells were stained with either 4’,6-diamidino-2-phenylindole (DAPI) (50 μM, for 30 min) or Syto9 (6 μM, for 20 min) (Molecular Probes, Eugene, Oregon, USA).

**1.4** **Microscopic analysis**

***Confocal laser scanning microscopy***

Microscopic analysis was performed on cells prepared by FISH with a white light laser confocal microscope (Leica TCS SP8 X) fitted with a 405 nm diode laser (Leica Microsystems, Kista, Sweden). Excitation (Ex.) and emission (Em.) details applied are as follows: DAPI (Ex. 405 nm; Em. 440-615 nm); Syto9 (Ex. 485 nm; Em. 490-550 nm); FLUOS (Ex. 492 nm; Em. 500-555 nm); Cy3 (Ex. 554 nm; Em. 565-650 nm); Cy5 (Ex. 649 nm; Em. 660-695 nm).

***Raman spectroscopy***

To locate the Acetothermia cells for Raman analysis, FISH was conducted on optically polished CaF_2_ Raman windows (Crystran, UK) by using the newly designed OP1 probes labelled with Cy3 (**Table S4**). Once the cells were located, the fluorescence of Cy3 was bleached by keeping the Raman laser on the target cell for 5 min. Raman spectra from single cells of Acetothermia were obtained using a Horiba LabRam HR 800 Evolution (Jobin Yvon – France) equipped with a Torus MPC 3000 (UK) 532 nm 341 mW solid-state semiconductor laser. Prior to all measurements, the Raman microspectrometer was calibrated to the first order Raman signal of Silicon occurring at 520.7 cm^-1^. The CaF_2_ Raman substrate also contains a single sharp Raman marker at 321 cm^-1^, which serves as an internal reference point in every spectrum. The incident laser power density on the sample was attenuated down to 2.1 mW/μm^2^ using a set of neutral density (ND) filters.

The Raman system is equipped with an in-built Olympus (model BX-41) fluorescence microscope. A 50x, 0.75 numerical aperture dry objective (Olympus M Plan Achromat- Japan), with a working distance of 0.38 mm was used throughout the work. A diffraction grating of 600 mm/groove was used, and the Raman spectra collected spanned the wavenumber region of 200 cm^-1^ to 1800 cm^-1^. The slit width of the Raman spectrometer and the confocal pinhole diameter were set to 100 μm and 150 μm, respectively. Raman microspectrometer operation and subsequent processing of spectra were conducted using LabSpec version 6.4 software (Horiba Scientific, France).

***Atomic force microscopy (AFM)***

The combined optical and atomic force microscopy experiments were carried out with a sample stained with 10 µM Syto9 in PBS solution. A JPK Nanowizard IV system (Berlin, Germany) on an inverted Zeiss Axiovert 200M epifluorescence microscope was used, with a 63x oil immersion optical lens (Zess Plan-Apochromat, NA 1.4) and Zeiss filter set 10 (Ex. 450-490 nm, Em. 525-565 nm). This AFM setup was used in the QI™ mode, which is a dynamic nanomechanical mapping (DNM) method. This DNM method can simultaneously provide height channels for morphology, and force spectroscopy based information, i.e. adhesion channel and force-distance curves. Although DNM methods are often used for accessing mechanical and physicochemical properties of the sample, they are also employed for high-resolution imaging due to their capability to directly control the tip-sample interaction forces below nanoNewton level. In this work, DNM was employed for advanced imaging, and the scans were acquired with a soft cantilever, namely Scanasyst-Air (Bruker). Nominal values for the cantilever’s resonance frequency and the spring constant are 70 kHz and 0.4 N/m, respectively. The operation parameters such as set point and Z length were varied to optimize the scan for the highest resolution and to minimize the risk of damaging the tip and the sample. The pixel time was kept at 30 ms. Z range was set to 15 µm, and the images were initially acquired with 256×256 px, then with 512×512 px, if possible. All DNM experiments were carried out in air under room conditions.

# **Supplementary results**

## 2.1 Fluorescence *in* situ Hybridization (FISH)

### ***2.1.1*** ***Probes designed for FISH***

FISH probes were designed to target defined clades within the Acetothermia phylum. The OP1-702 and OP1-838 probes were used to cover the proposed novel genus containing Acetothermia sp. Ran1. Both probes cover all sequences classified to the genus, without mis-targeting any outside the cluster (**Figure S4**). As no pure cultures with less than two mismatches to any of the probes were available, probes were optimized with fixed sludge samples originating from an anaerobic digester at Randers WWTP. Members of Acetothermia phylum were found to be abundant in these samples by DNA sequencing (**Figure 1**).

The OP1-702 probe requires the helper probe OP1-702_H1, but not OP1-702_H2, to obtain a detectable signal. The OP1-838 probe does not require either of the designed helper probes, although the inclusion of the OP1-838_H2 probe gives a noticeable increase in signal and is therefore recommended for use (**Table S4**). The OP1-702 had superior signal to the OP1-838 probe, and we recommend applying them both with different fluorochromes for increased confidence in their specificity. Good overlap for these probes was observed when applied on the PFA-fixed digester sludge (**Figure S5**) from Randers.

#### **2.1.2 Observation of prosthecae with Ethanol- and PFA-fixed samples**

For FISH analyses with ethanol fixed biomass, patchy signal was observed for the appendages of the Acetothermia cells compared to the strong, even signal from the rod-shaped body. The lower signal for the prosthecae compared to the cell body probably indicates a lower level of ribosomes in the former. FISH signal was only observed for the rod-shaped body with PFA fixed cells (**Figure S6**). The varied result with different fixatives likely reflects different membrane properties of the cell body and the appendages. Ethanol based fixation dehydrate cells, causing proteins to denature and precipitate in situ, also increasing the permeability of the cell. Paraformaldehyde causes covalent cross-links between molecules, effectively gluing them together into an insoluble meshwork and leaving the membrane intact, resulting in less permeability. As the width of the prosthecae (0.256±0.004 µm) is about half of the main rod body (0.46±0.03 µm), the prosthecae have higher surface to volume ratio (15.6 μm^-1^) compared with the body (9.6 μm^-1^), which could make the cell membrane more rigid and difficult to penetrate in the prosthecae when fixed with PFA. This effect may not be as pronounced for the ethanol fixed cells due to a generally higher cell permeability. Other differences in membrane properties for the prosthecae and cell body are also potential contributing factors to the response to different fixatives and further work is required to elucidate the specific reasons.

## 2.2 Optimizing PCR primers for amplicon sequencing

The choice of PCR primers can have a pronounced bias on the microbial composition observed (Albertsen *et al.*, 2015). Three common 16S rRNA gene amplicon primer pairs were evaluated on samples from a digester containing Acetothermia, and metagenomes of the same samples derived from primer-independent shotgun sequencing were used as references (**Figure** **S2**). Results showed that, only the 515F/806R (Caporaso *et al.*, 2011) primer pair, which targets V4 region of the 16S rRNA gene, was able to amplify Acetothermia-related 16S rRNA genes and provide an estimate of Acetothermia relative abundance in the samples. To ensure that the primer pair would be able to target all Acetothermia, we compared the primer sequences to Acetothermia sequences in the SILVA databases. It was found that 67.4% of the sequences contained a single mismatch to the 806R primer. However, this could be alleviated by increasing the degeneracy of the primer at a single position, which generated a modified primer m806R. This modification did not affect the overall community structure of the samples tested (**Figure** **S2**), therefore, m806R was used for the survey.

## 2.3 Metabolic model

### ***2.3.1 Solute uptake***

Several ABC transporters reflecting the heterotrophic metabolism of this microorganism are found in the genome, including those for importing amino acids (*livF,G,H,M*), peptides (*oppA,B,C,D,E,F* and *dppA,B,C,D,E,F*; the character in red indicates missing gene code), Glycerol-3-phosphate (*ugpA,B,C,D,E*), maltose (*malE,F,G,K*), ribose (*rbsA,B,C*), alpha-glucoside (*aglE,F,G*), and probably a carbohydrate transporter, whose specificity is unknown (T2 in **Supplementary Data Set2**).

Besides, ion transport systems for the acquisition of K^+^ (*trkA,H*), Mn^2+^ (*mntA,C*), Zn^2+^ (*zupT*), Fe^2+^ (*feoA/B*), cobalamine /Fe^3+^-siderophore, phosphate/phosphonate (*phnA,B,C*), sulfonate/nitrate/taurine (*sufA,B,C*), and thiamine (*thiB,P*) were found in the genome. The presence of these transporters is expected to contribute to efficient cation uptake to support cell survival and growth. Transporters for Mg^2+^ (*mgtE*), NH_4_^+^ (*amt*) were not found.

### ***2.3.2 Sugar metabolism***

Ran1 harbors an extracellular cellulase (HyG7 in **Supplementary Data Set2**), which can catalyze the endohydrolysis of (1->4)-beta-D-glucosidic linkages in cellulose, lichenin, and cereal beta-D-glucans (Ec. 3.1.2.4) and an extracellular glycoside hydrolase family protein (HyG5 in **Supplementary Data Set2**), demonstrating that it has some extracellular polysaccharide hydrolysis ability. The putative alpha-glucosidases (HyG2,4 in **Supplementary Data Set2**) can hydrolyze (1->4)-alpha-D-glucosidic linkages in starch, glycogen, and other related polysaccharides to remove successive maltose units from the non-reducing ends of the chains. This reaction can provide targets for the MalEFG transporters if the alpha-glucosidases are secreted. Nevertheless, there is no clear evidence for the subcellular localization of the enzymes. As the genome encodes a complete glycogen pathway (SS1-5 in **Supplementary Data Set2**), the possibility of hydrolyzing glycogen to dextrin and maltose cannot be ruled out for the glucosidases if they are present inside the cell. The imported maltose can be hydrolyzed to glucose by an amylomaltase (SS7 in **Supplementary Data Set2**), which then enters the glycolysis pathway. All the genes involved in the Embden-Meyerhof-Parnas pathway (Gly1-11 in **Supplementary Data Set2**) and gluconeogenesis (Glu12-16 in **Supplementary Data Set2**) were found, except a glucose-6-phosphatase catalyzing the reaction: D-glucose 6-phosphate + H_2_O => D-glucose + phosphate.

Three sugar kinases: hexokinase (Gly1 in **Supplementary Data Set2**), ribokinase (P8 in **Supplementary Data Set2**), putative fructose-specific sugar kinase (SS9 in **Supplementary Data Set2**) were detected, implying this bacterium can directly utilize a few hexoses and pentoses as carbon source, through the Embden-Meyerhof-Parnas/gluconeogenesis pathways, and the non-oxidative pentose phosphate pathway, which was completely encoded in the genome (P1-8 in **Supplementary Data Set2**).

### ***2.3.3 Glycerol metabolism***

Glycerol or glycerol-3-phosphate are probably used for anabolic pathways, as glycero-3-phosphate dehydrogenase, the key enzyme for glycerol degradation, was missing (**Supplementary Data Set2**).

### ***2.3.4 Incomplete tricarboxylic acid (TCA) pathway***

The genome encoded an incomplete tricarboxylic acid (TCA) pathway as a succinate dehydrogenase (*sdhA,B,C,D*)/fumarate reductase (*frdA,B,C,D*) complex was not annotated. Through the incomplete TCA cycle, all the intermediates, including oxaloacetate, fumarate, succinyl-CoA, α-ketoglutarate, and citrate can be synthesized from pyruvate and acetyl-CoA. The conversion between fumarate and succinate catalyzed by the membrane-binding *sdh*/*frd* complex is lacking, which is a key step linked to the electron transport chain (ETC) and oxidative respiration. This was also observed for the ‘Acetothermia bacterium 64_32’ genome by Hu et al. (Hu *et al.*, 2016) . Considering that no complete ETC for aerobic or anaerobic respiration was found for Ran1, the incomplete TCA pathway may serve as source of biosynthetic precursors for building blocks in the reductive direction at the expense of reducing power and incorporation of CO_2_ as in methanogens and other anaerobic bacteria (Rosenberg *et al.*, 2013; Nobu *et al.*, 2016) . In addition, oxaloacetate and α-ketoglutarate from this incomplete TCA cycle are also intermediate metabolites during amino acids catabolism, as well as pyruvate, 3-phosphoglycerate, and other metabolites from the glycolytic and PPP pathways. The enzymes can therefore act as the entry points of amino acids into the metabolic mainstream (Bridger *et al.*, 2012; Adams *et al.*, 2001; Fukui *et al.*, 2005).

### ***2.3.5 Amino acid metabolism***

#### Catabolism

8.8% (114 in 1290) of the genes with COGs annotation were classified into the class of amino acid transport and metabolism, suggesting that amino acid metabolism is an important metabolic process in this organism. Five types of ABC transporters (35 in 1324 CDSs) were encoded for uptake of polar amino acids, branched-chain amino acids, oligopeptides, dipeptides, peptide/nickel from the environments (**Supplementary Data Set2**).

Peptides and amino acids are hydrolytic products of proteins from the biological and primary sludge in digesters, catalyzed by extracellular proteases and peptidases. For Ran1, even though 31 proteases and peptidases were identified, most of them are cytoplasmic proteins, and only one extracellular peptidase and one periplasmic protease DegP (also known as HtrA) were encoded. The bacterium thus has genetic potential for some extracellular protein hydrolytic activity. Ran1 encoded genes for catabolizing at least 13 of the 22 amino acids (**Supplementary Data Set2, Figure S10, S11**), which is comparable to the known proteolytic bacterium *Deinococcus proteolyticus* and the peptide-utilizing archaea *Pyrococcus furiosus* and *Thermococcus kodakarensis* (Copeland *et al.*, 2012).

#### Anabolism

We detected complete synthetic pathways only for glutamate, glutamine, cysteine, glycine, and aspartate, and incomplete pathways for alanine, serine, threonine, tryptophan, and leucine (**Supplementary Data Set2**). Ran1 is therefore dependent on the uptake of amino acids which it cannot synthesize from the environment to sustain growth.

### ***2.3.6 Hdr gene in sulfur metabolism***

Ran1 does not encode pathways for sulphate reduction. However, the complex formed by the electron-bifurcating heterodisulfide reductase (Hdr A-C) and the methyl viologen reducing hydrogenase (Mvh D,G,A) could function as a polysulfide/disulfide oxidoreductase (**Figure** **S12**), as proposed for other anaerobic bacteria (Nobu *et al.*, 2015a; Kirkegaard *et al.*, 2016). The Hdr gene in methanogens acts on the anaerobic respiration using disulfide CoM-S-S-CoB as the final electron acceptor and also catalyzes reduction of fumarate to succinate in the reductive TCA metabolism concomitant with regeneration of CoM-S-S-CoB from the 2 thiol-coenzymes (CoMSH and CoBSH) (Hedderich *et al.*, 1999).

Therefore, Ran1 might use polysulfide as an alternative electron acceptor to produce hydrogen sulphide (H_2_S) – thus contributing to the turnover of sulfur and production of H_2_S.

### ***2.3.7 Stress response***

Like other anaerobes (Hyun *et al.*, 2008; Fukui *et al.*, 2005), Acetothermia sp. Ran1 encodes several genes to protect it against oxidative stress. Superoxide reductase (*sor*) and ruberythrin (*rbr*) catalyze detoxification of the superoxide anion (O_2_^●-^) and peroxide (H_2_O_2_), using rubredoxin (rub) as electron carrier(Brines and Kovacs, 2007). The reduced rubredoxin is regenerated through the NAD(P)H-rubredoxin oxidoreductase (NROR). The cytoplasmic thioredoxins (trx) have low redox potentials and are involved in maintaining the reducing status of the cytoplasm (Erlendsson *et al.*, 2003). They act as electron donors to peroxidases (prx) and [ribonucleotide reductase](https://en.wikipedia.org/wiki/Ribonucleotide_reductase) and are kept in the reduced state by [thioredoxin reductase](https://en.wikipedia.org/wiki/Thioredoxin_reductase) (trxB) in a NADPH-dependent reaction (Erlendsson *et al.*, 2003). G[lutaredoxins](https://en.wikipedia.org/wiki/Glutaredoxin) (grx) have similar functions to those of thioredoxins. In addition, a gene homolog to bacterioferritin (Cytochrome b1) was found, which functions in connection with iron uptake, storage, and supply, and detoxification of iron and dioxygen in cell redox-stress resistance (Carrondo *et al.*, 2003). Presence of these genes related to redox-stress resistance may allow Ran1 to survive under microaerobic conditions.

# **Supplementary figures**

**
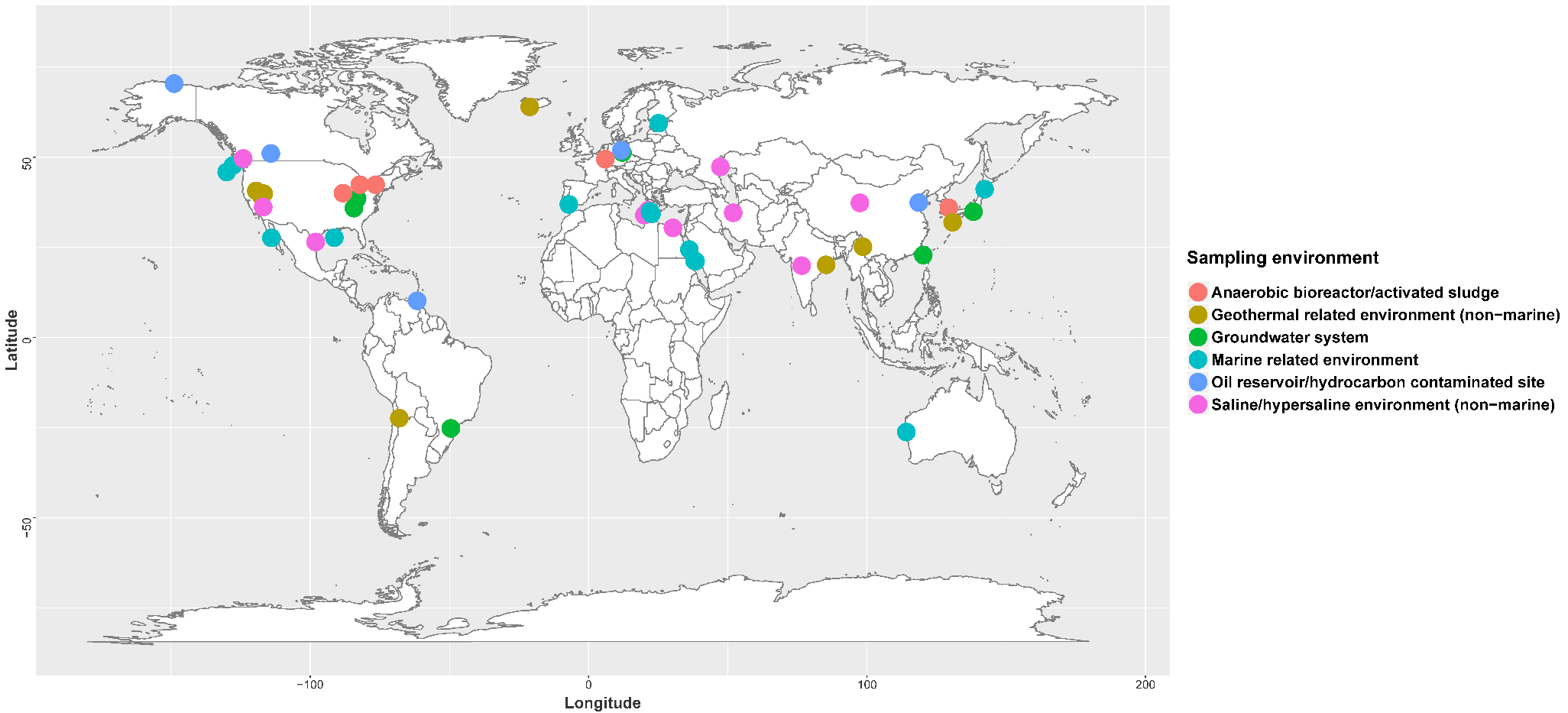
**

**Figure S1:** **Geographic location and sampling environments of selected Acetothermia sequences from the SILVA SSUParc 128 database.** The database contains in total 1209 sequences from 224 different isolation sources. 70 selected sequences from 51 studies were used in this figure, the study details of which can be found in publications (**Supplementary Data Set3**). To generate this figure, the Genbank IDs of the sequences classified as Acetothermia in SILVA (Quast *et al.*, 2013) were used to download the corresponding Genbank files. The fields “isolation source”, “PUBMED” and “lat_lon” were extracted to classify the sequences as originating from different types of environments. Sequences derived from engineered systems originated from 6 studies, including anaerobic bioreactors treating wastewater (Nobu *et al.*, 2015b; Perkins *et al.*, 2011) and organic wastes (Goux *et al.*, 2015; Chaganti *et al.*, 2012; Goux *et al.*, 2016), and a fixed-film activated sludge reactor (Kwon *et al.*, 2010). Most of the sequences originated from natural systems, including saline/brackish water, marine/lake/river sediments, subseafloor fluids/mud volcano, salt pan/saltworks, oil reservoir/hydrocarbon contaminated sites, hot spring/geothermal water stream/sediment, ground water/aquifer, peatland, biofilm (**Supplementary Data Set3**). Generally, the survey shows that members of candidate phylum Acetothermia are globally dispersed and typically associated with anaerobic environments.


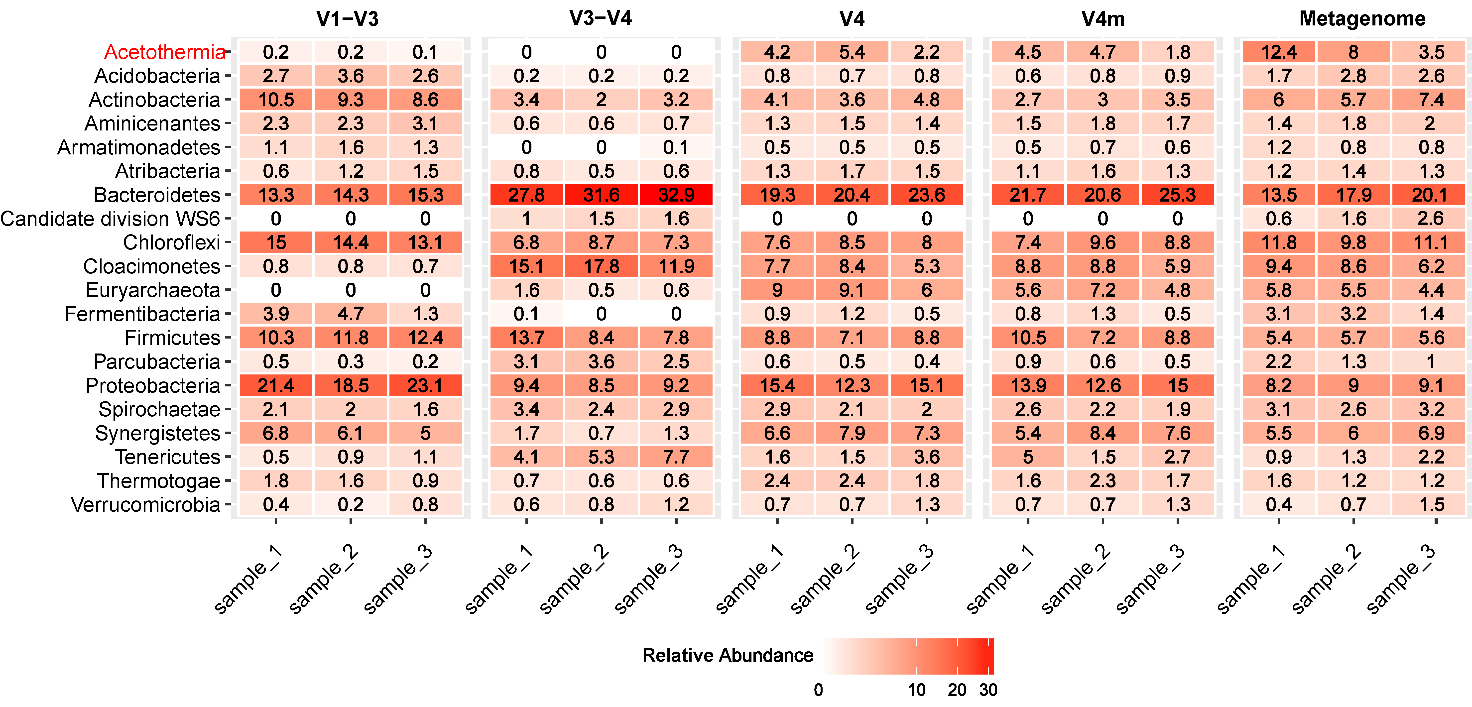


**Figure S2: Effect of universal PCR primers on detection of Acetothermia with amplicon sequencing.** Three commonly used universal primer pairs (V1-V3, V3-V4, V4) and a modified pair (V4m) were tested with amplicon sequencing method (Albertsen *et al.*, 2015) on sample_1, 2, 3 taken at 3 different time points from a sludge digester at Randers wastewater treatment plant (**Supplementary Data Set1**). These primers included 27F/534R (Consortium *et al.*, 2012), Bakt_341F/Bakt_805R (Herlemann *et al.*, 2011; Klindworth *et al.*, 2013), 515F/806R (Caporaso *et al.*, 2012) and its modified version 515F/m806R (**Supplementary Data Set1**), respectively targeting the V1-V3, V3-V4, and V4 regions of the prokaryotic 16S rRNA genes. PCR-independent shotgun sequencing was performed on the same samples, resulting in generation of 3 metagenomes. 16S rRNA gene sequences produced from amplicon sequencing or extracted from metagenomes were processed using methods described by Albertsen *et al*. (2015). Microbial compositions were compared at phylum level with metagenomes as reference. The 20 most abundant phyla detected from the metagenomes were shown and sorted alphabetically, which was performed in R (version 3.4.1) through the Rstudio IDE (http://www.rstudio.com/). Ampvis package (v. 1.27.0) was used to visualize the results. Numbers in the strips of the heatmap represent the percentage values of reads assigned to different phyla.

**
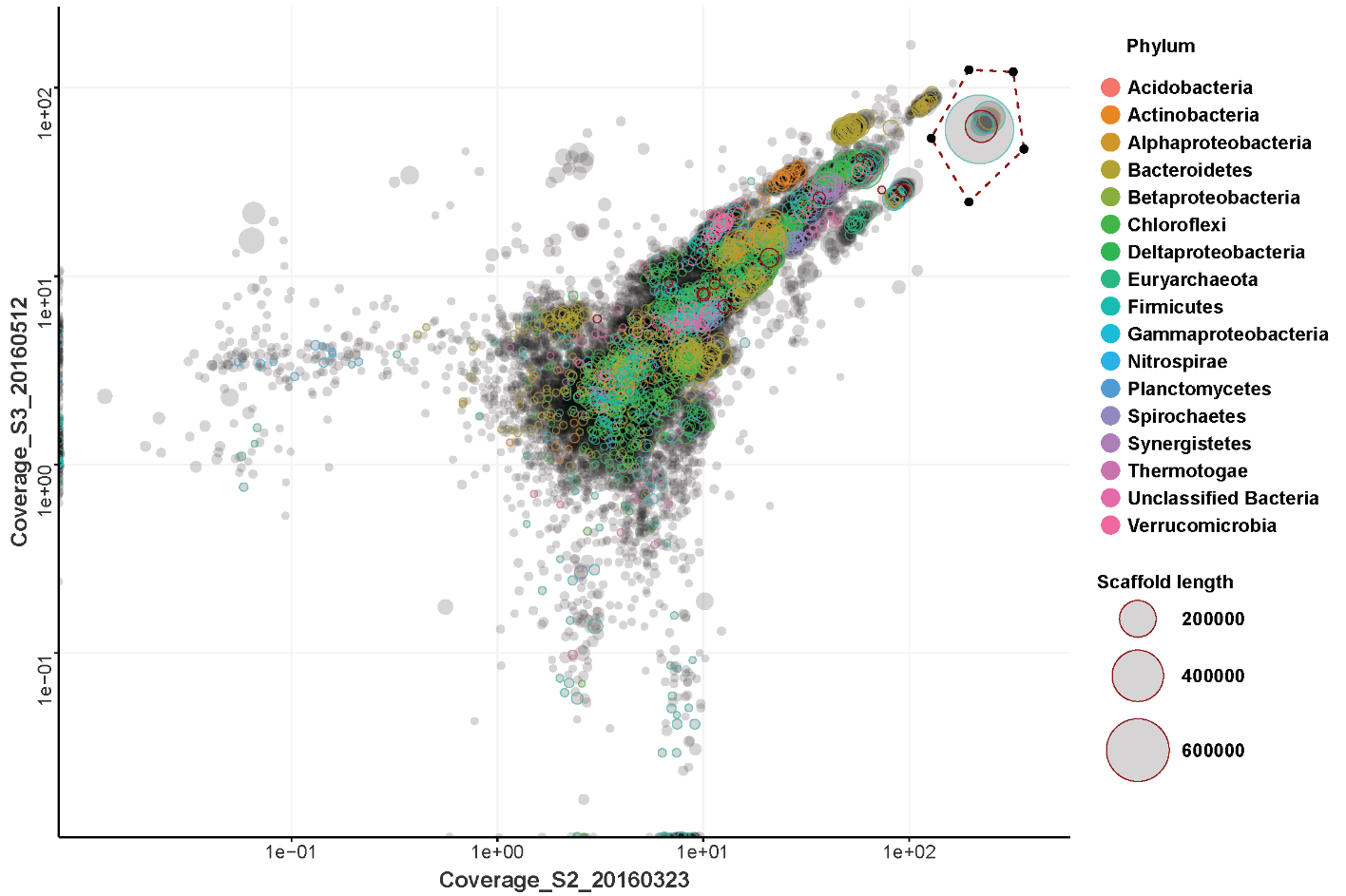
**

**Figure S3: Extraction of the** **Acetothermia sp. Ran1 genome bin from the metagenome scaffolds by differential read coverage** using biomass samples (S2 and S3) taken from the anaerobic digester at Randers wastewater treatment plant (on 2016-03-23 and 2016-05-12). Circles represent scaffolds, colored according to phylum-level taxonomic affiliation. Only scaffolds ≥5000 bp are shown. The axes are log scaled. The Acetothermia sp. Ran1 genome bin is enclosed by the pentagon.


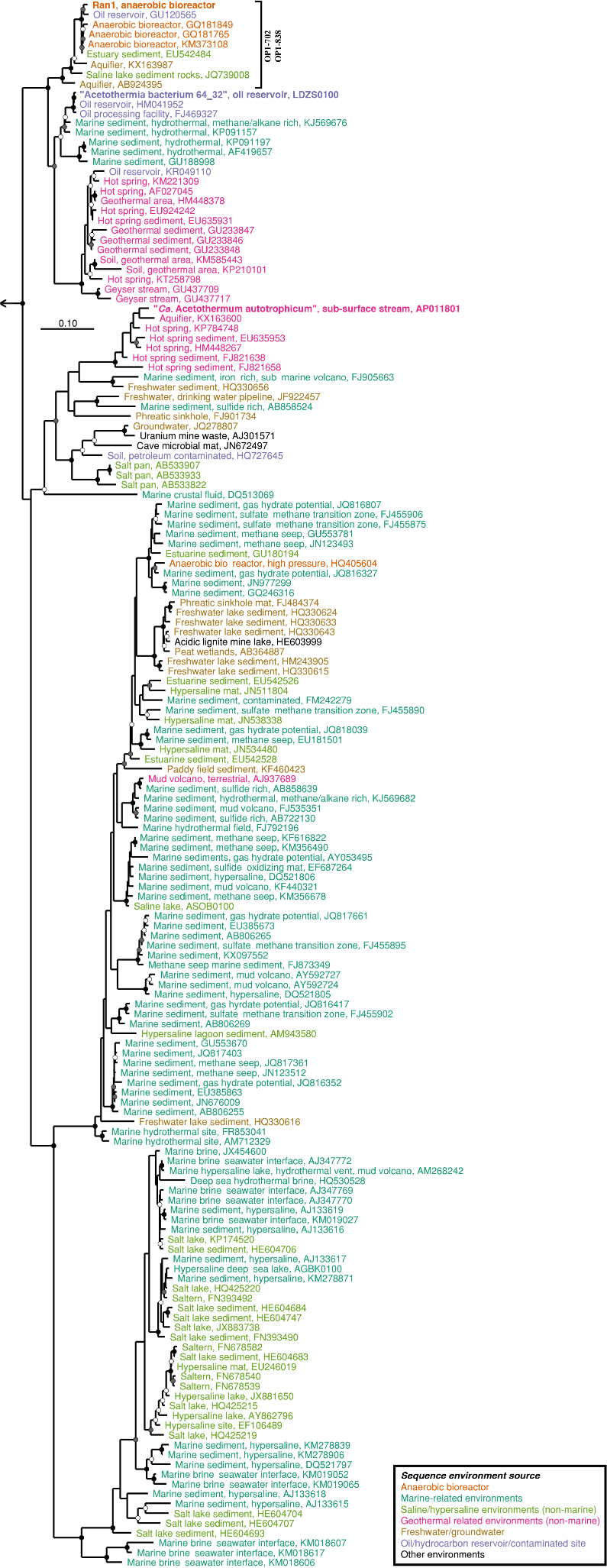


**Figure S4: Maximum-likelihood (PhyML) 16S rRNA gene phylogenetic tree of sequences classified to Acetothermia (**SILVA SSURef NR 99, Release 128**)**. The alignment used for the tree applied a 20% conservational filter to remove hypervariable positions, giving 1120 aligned positions. Sequences are colored according to their source environment. Proposed phylogenetic classification of the novel genus and coverage of the newly-designed FISH probes are indicated with a black bracket. Bootstrap values from 100 re-samplings are indicated for branches with >50% (white dot), 50-70% (grey dot) and >90% (black dot) support. Species of the phylum Thermotogae were used as the outgroup. The scale bar represents substitutions per nucleotide base.

**
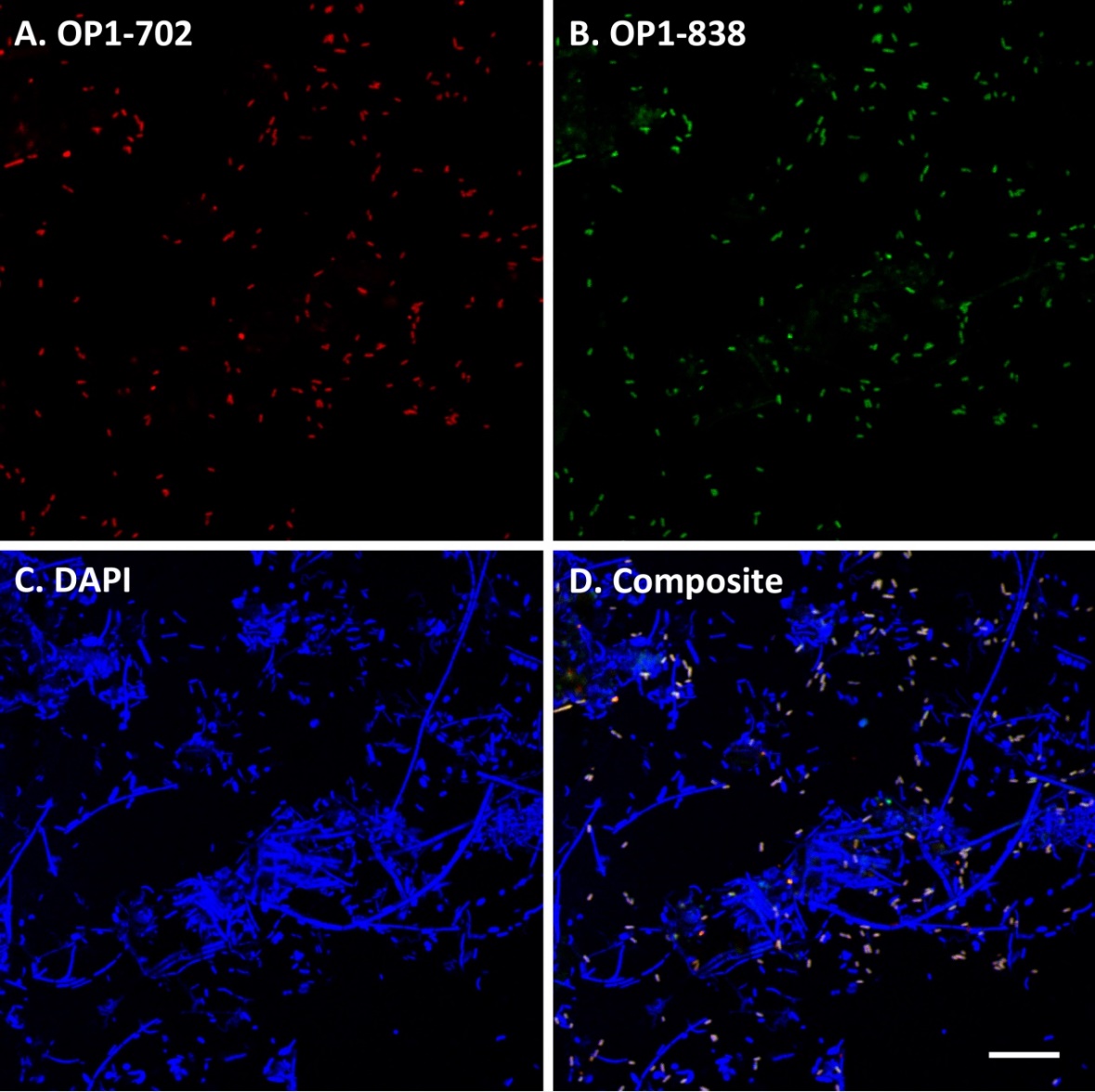
**

**Figure S5: Fluorescence micrographs of the Acetothermia cells showing overlap of signals from the OP1-702 and OP1-838 probes**. (A) FISH image with the OP1-702 probe (Cy3, red); (B) FISH image with the OP1-838 probe (Cy5, green); (C) DAPI stained (blue); (D) Composite image of A, B, and C, Acetothermia cells appear white (OP1-702 red + OP1-838 green + DAPI blue = white), and other cells appear blue. Biomass was taken from anaerobic digester sludge at Randers wastewater treatment plant and fixed with PFA. Scale bar indicates 10 μm.

**
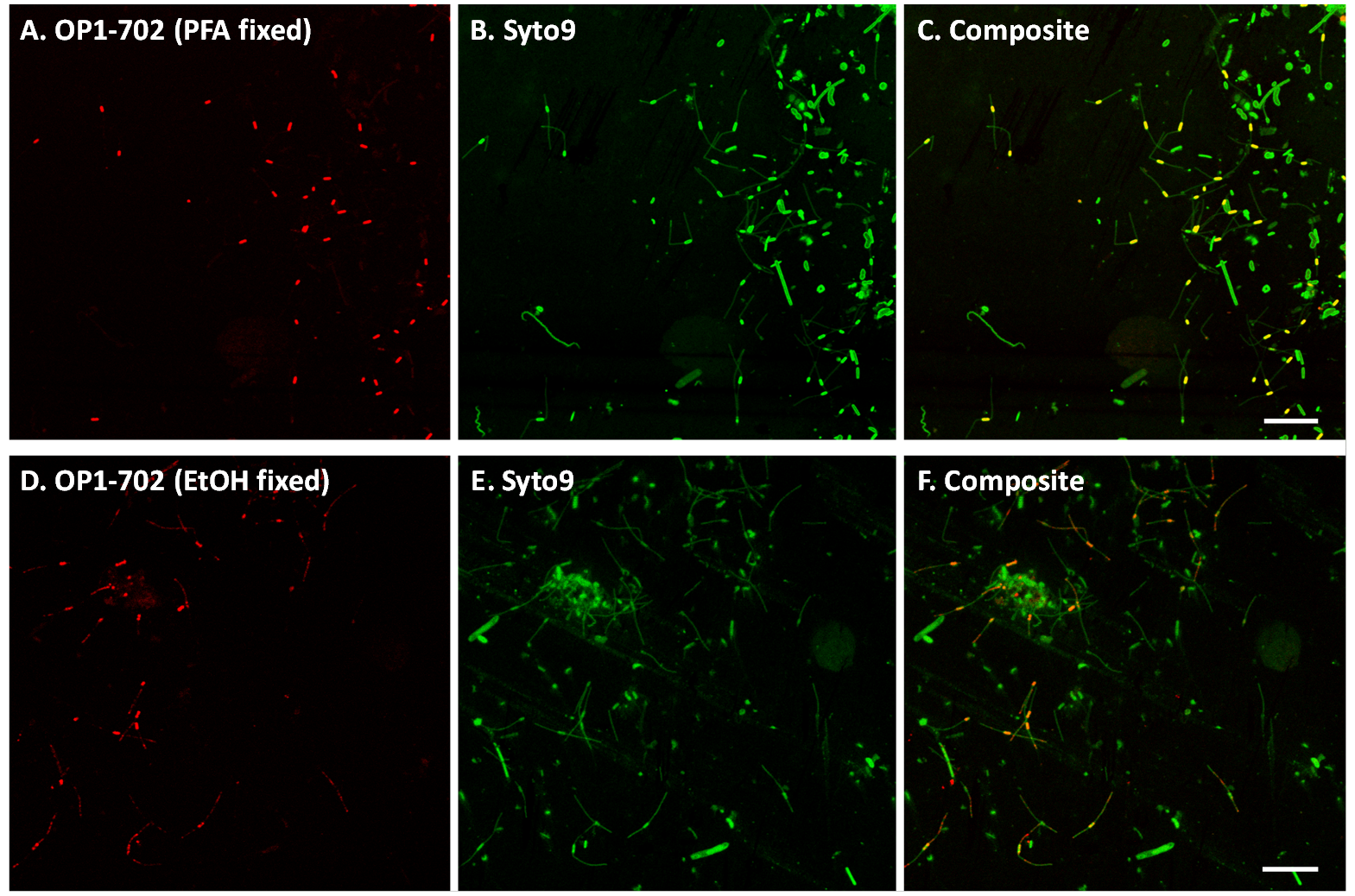
**

**Figure S6: Fluorescence micrographs of the Acetothermia cells fixed with PFA and ethanol.** (A, D) FISH images with the OP1-702 probe (Cy3, red); (B, E) Syto9 stained (green); (C, F) Composite images of A and B, and D and E, respectively. (A, B, C) PFA fixed biomass; (D, E, F) Ethanol fixed biomass. FISH signal for the cellular appendages is only observed for the ethanol fixed biomass. Biomass was taken from anaerobic digester sludge at Randers wastewater treatment plant. Scale bar represents 10 μm.


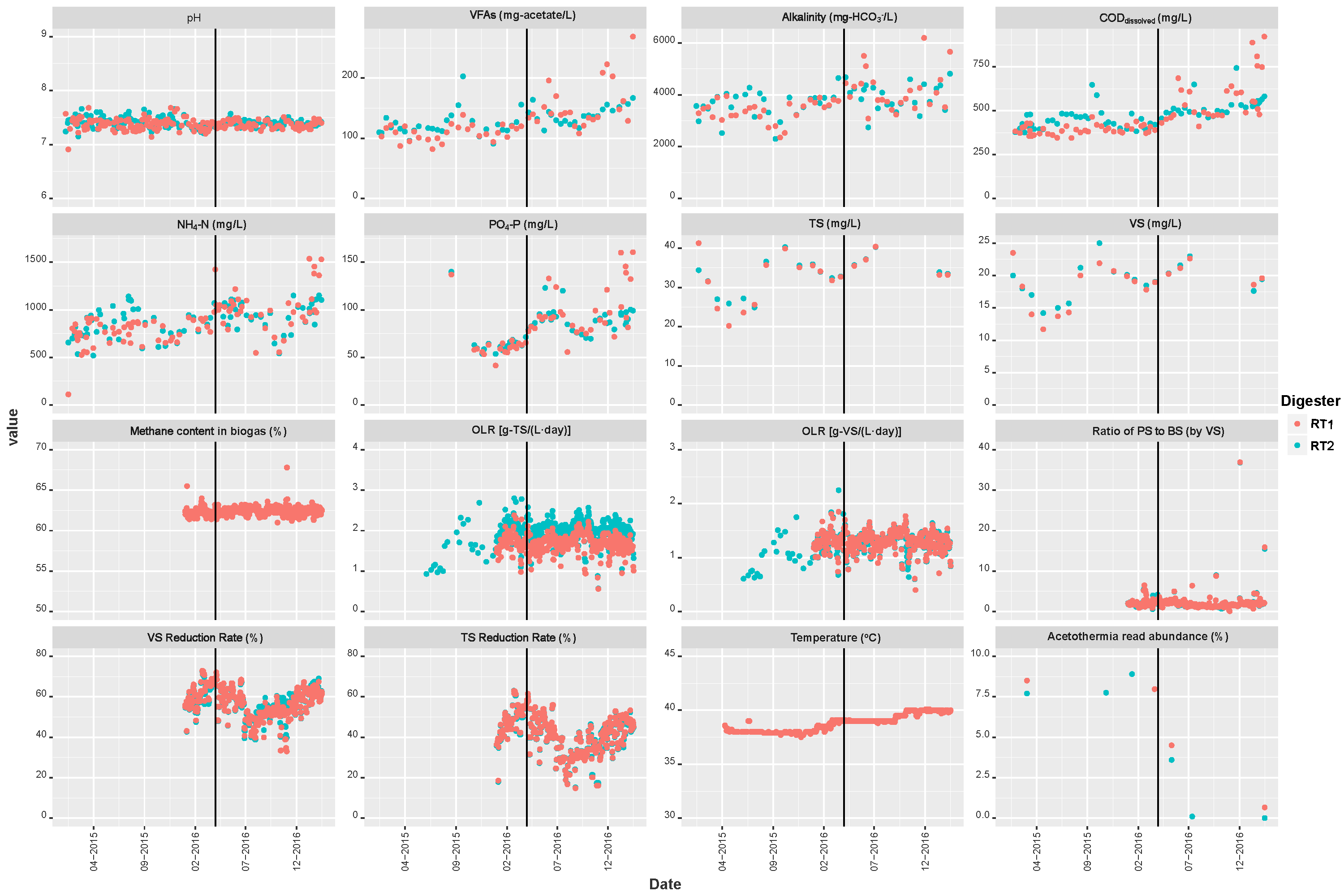


**Figure S7: Operational and performance conditions of two anaerobic digesters RT1 and RT2 at Randers WWTP, with relative abundance of Acetothermia at different periods**. X axis labels represent year and month of sampling time. TS = Total Solids; VS = Volatile Solids; OLR = Organic Loading Rate; COD = Chemical Oxygen Demand; PO_4_-P = orthophosphate, inorganic phosphate phosphorus; VFSs = Volatile Fatty Acids; PS = Primary Sludge; BS = Biological Sludge. The black vertical line indicates the time point at which relative abundance of Acetothermia decreased to a lower level.


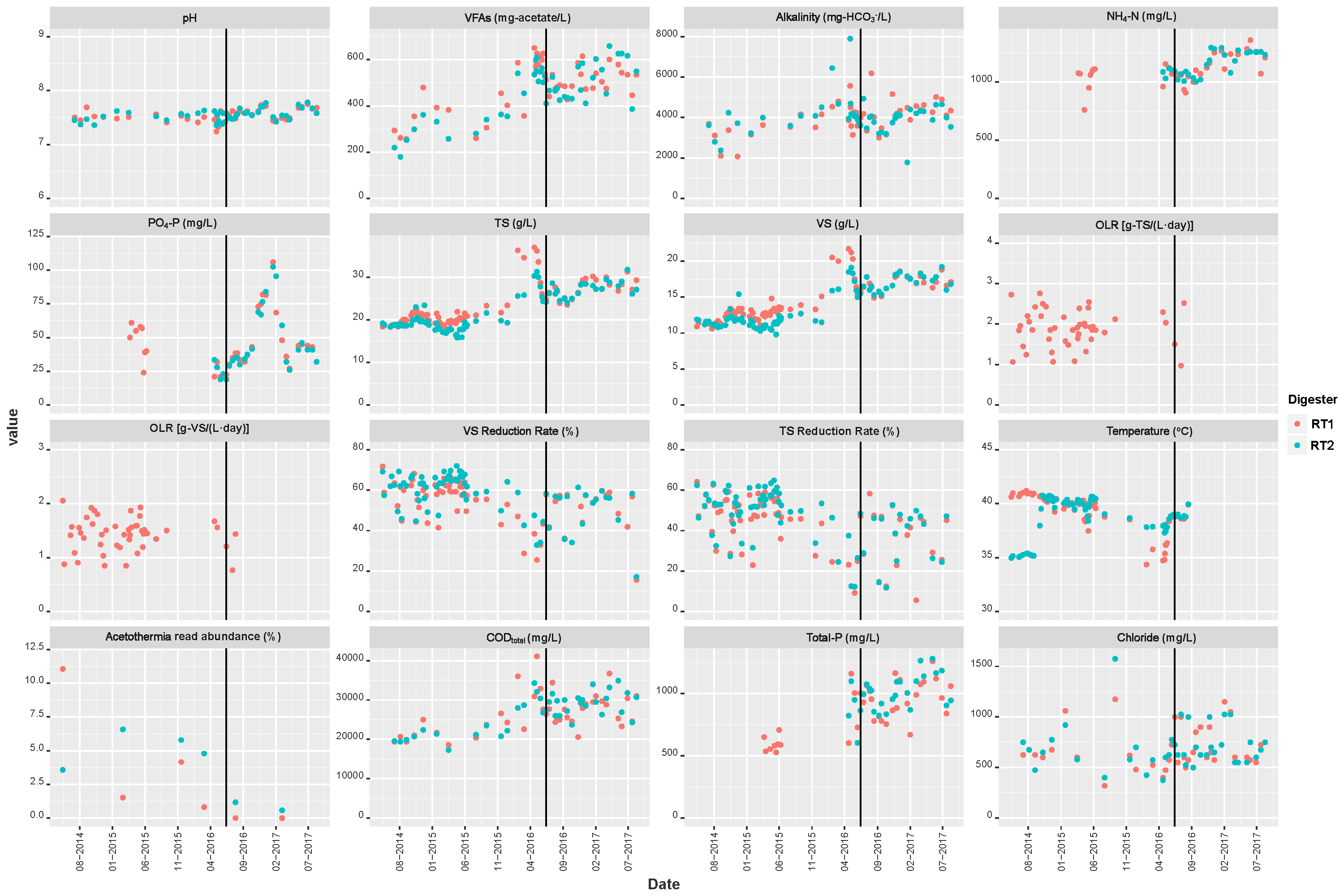


**Figure S8: Operational and performance conditions of digesters RT1 and RT2 at Esbjerg WWTP, with relative abundance of Acetothermia at different periods**. X axis labels represent year and month of sampling time. TS = Total Solids; VS = Volatile Solids; OLR = Organic Loading Rate; COD = Chemical Oxygen Demand; Total-P = inorganic and organic phosphate phosphorus; VFSs = Volatile Fatty Acids; PS = Primary Sludge; BS = Biological Sludge. The black vertical line indicates the time point at which relative abundance of Acetothermia decreased to a lower level.


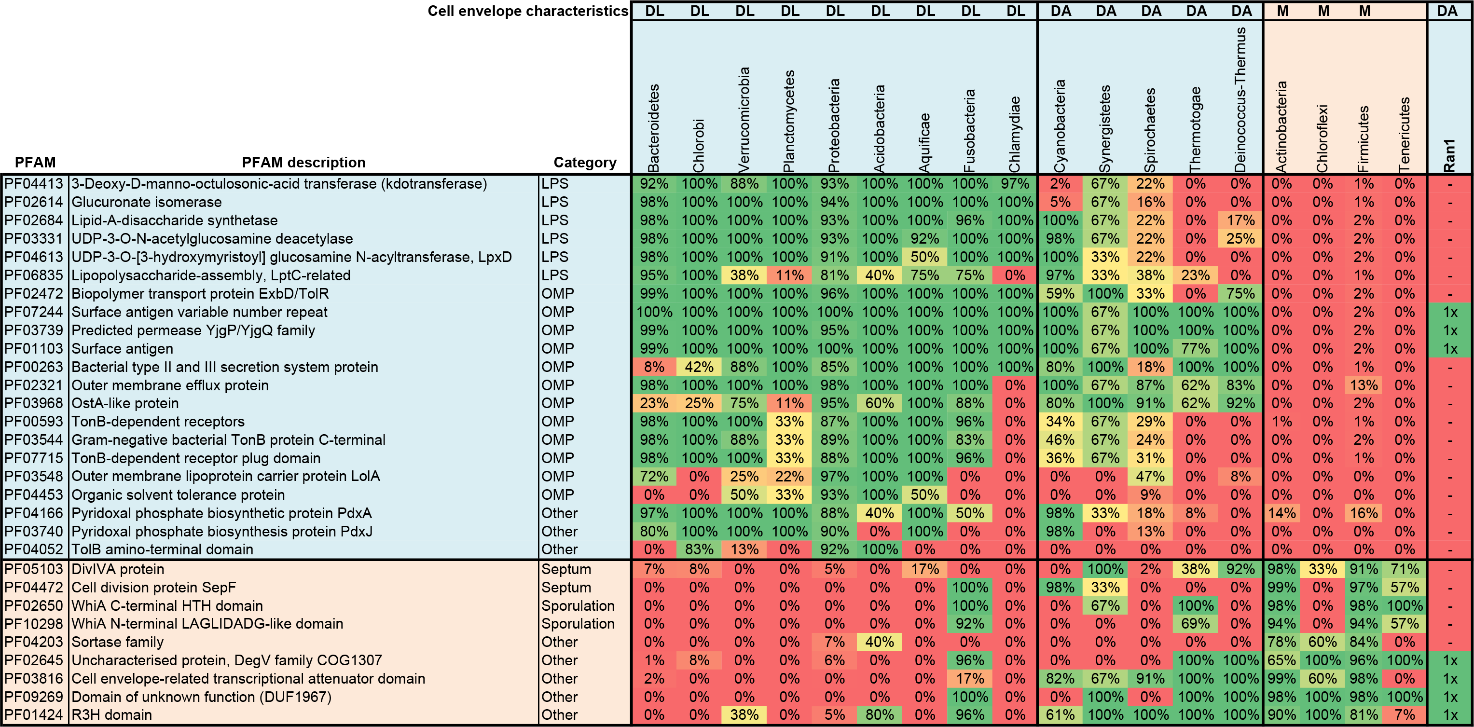


**Figure S9:** **Cell envelope classification of Ran1**. Analysis was based on a search into the genome for genes encoding PFAM proteins (Finn *et al.*, 2016) that are specific to archetypical mono- (M) or diderm bacteria with lipopolysaccharides (DL) or atypical diderm bacteria (DA) as detailed previously by Albertsen *et al.* (Albertsen *et al.*, 2013). These include proteins involved in lipopolysaccharide synthesis (LPS), outer membrane associated proteins (OMP), and proteins associated with septum formation and sporulation. The percentage prevalence of each PFAM is given for each listed phylum. Phylum level data is calculated based on all complete genomes in the IMG database (release 3.5) (Chen *et al.*, 2017). Only phyla represented by at least four complete genomes were included. The numbers shown in the column for Ran1 represent the number of hits for a given PFAM protein in the analyzed genome. The PFAM profile of Ran1 is atypical, but indicates a simple diderm cell envelope without LPS.


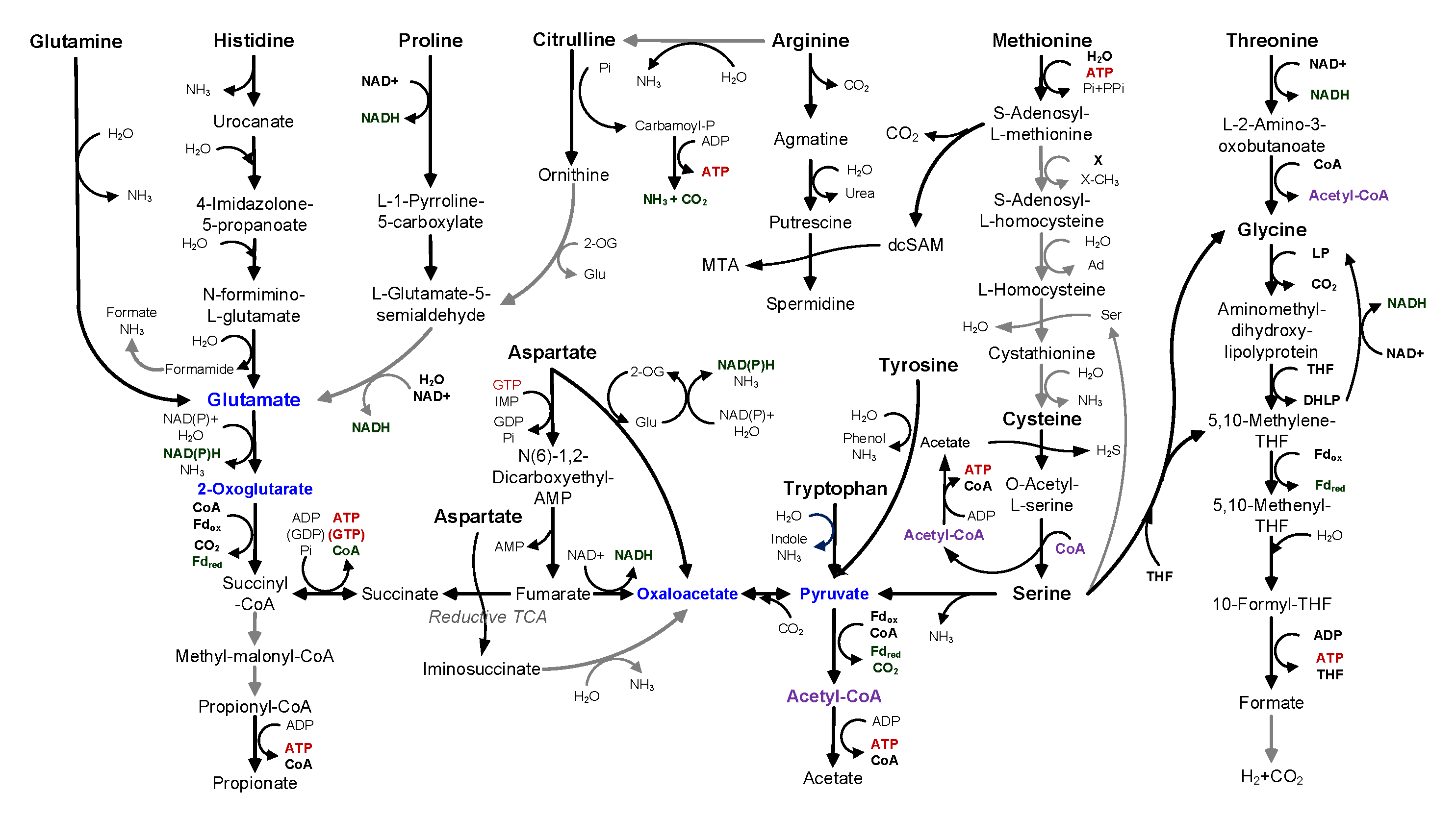


**Figure S10**: **Degradation pathways of amino acids (non-branched-chain) reconstructed from Ran1 genome**. The specific pathways that are encoded are denoted with black arrows. Grey arrows indicate missing genes. Degradation mechanisms of lysine, alanine, phenylalanine and asparagine are not detected in Ran1 genome, thus not shown in this figure. Co-reactants and co-products include adenosine triphosphate (ATP, in red), adenosine diphosphate (ADP), guanosine-5'-triphosphate (GTP, in red), [guanosine diphosphate](https://en.wikipedia.org/wiki/Guanosine_diphosphate) (GDP), inosine monophosphate (IMP), inorganic phosphate (Pi), [pyrophosphate](https://en.wikipedia.org/wiki/Pyrophosphate) (PPi), coenzyme A (CoA), tetrahydrofolate (THF), lipoylprotein (LP), 2-oxoglutarate (2-OG), glutamate (Glu), serine (Ser), adenosine (Ad), oxidized/reduced ferredoxin (Fd_ox_ and Fd_red_, in green), oxidized/reduced nicotinamide adenine dinucleotide phosphate (NADP^+^ and NADPH, in green), oxidized/reduced nicotinamide adenine dinucleotide (NAD+ and NADH, in green), S-adenosyl 3-(methylthio)propylamine (dcSAM), and 5'-S-methyl-5'-thioadenosine (MTA).


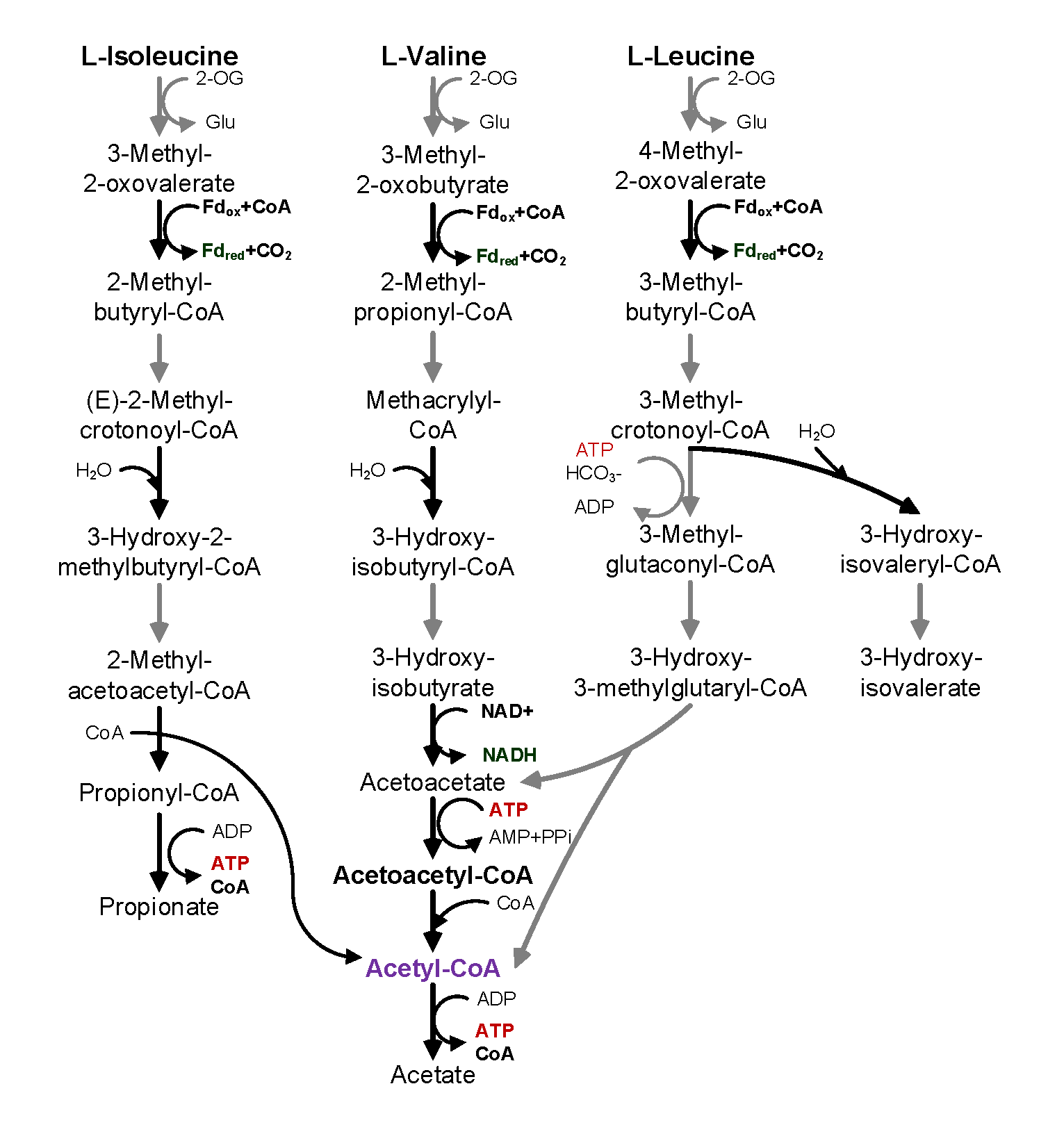


**Figure S11: Degradation pathways of branched-chain amino acids (leucine, valine, and isoleucine) reconstructed from Ran1 genome.** The specific pathways that are encoded are denoted with black arrows. Grey arrows indicate missing genes. Co-reactants and co-products include adenosine triphosphate (ATP, in red), adenosine diphosphate (ADP), adenosine monophosphate (AMP), [pyrophosphate](https://en.wikipedia.org/wiki/Pyrophosphate) (PPi), coenzyme A (CoA), 2-oxoglutarate (2-OG), glutamate (Glu), oxidized/reduced ferredoxin (Fd_ox_ and Fd_red_, in green), oxidized/reduced nicotinamide adenine dinucleotide (NAD^+^ and NADH, in green).


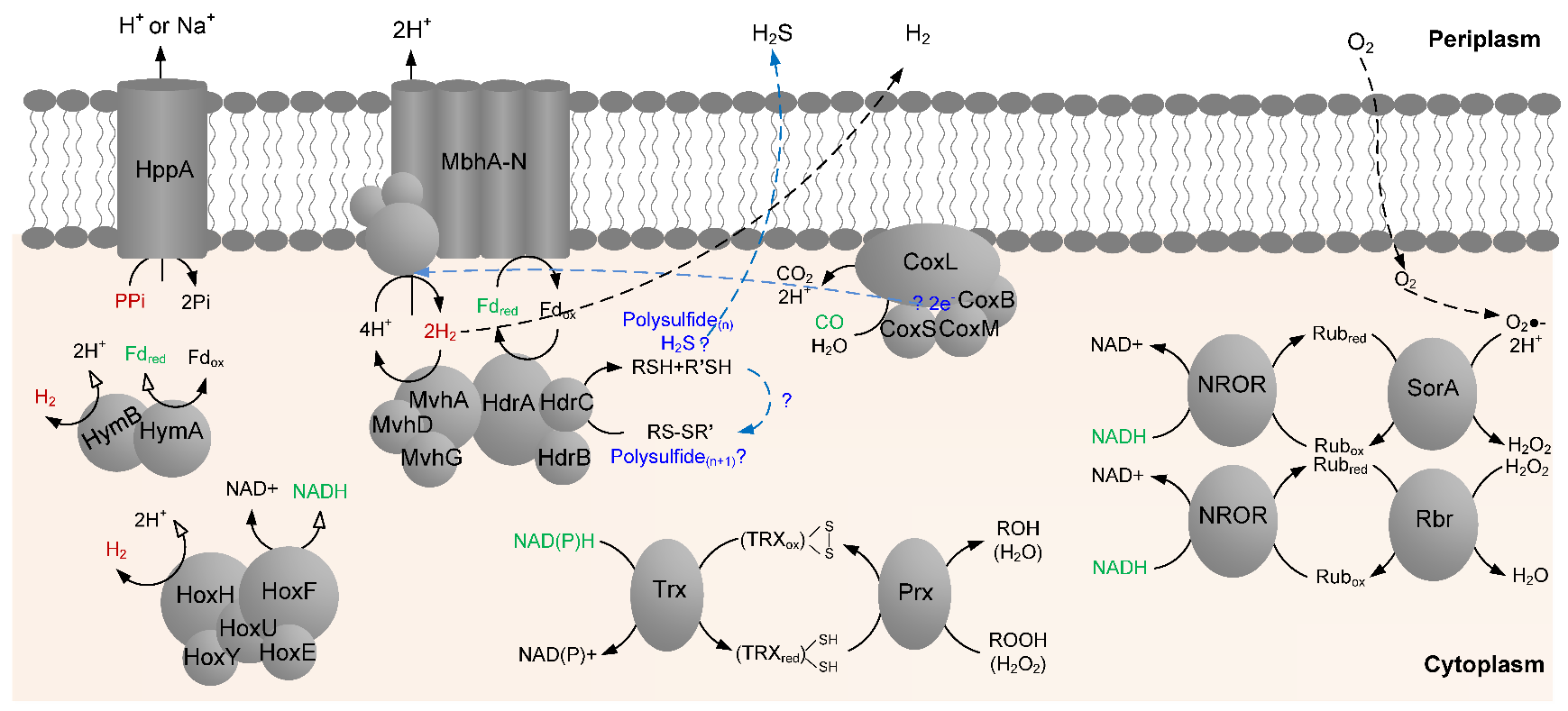


**Figure S12: Electron transport relevant to energy conservation and anti-oxidation mechanisms in Ran1**. Dash line arrows show hypothesized flux. Ran1 has putative genes for H_2_ production by an energy-conserving membrane-bound hydrogenase complex (MbhA-N). It can translocate protons across the membrane during electron transport to generate proton motive force (PMF), with the reduced ferredoxin as electron donor (Buckel and Thauer, 2013; Nobu *et al.*, 2015b). Alternatively, electrons could be donated from CO oxidation catalyzed by the membrane-associated carbon monoxide dehydrogenase complex (CoxLMS). This is a hypothesis, as the electron acceptor during CO oxidation is not clear. H_2_ can be recycled through the methyl viologen reducing hydrogenase (Mvh D,G,A), which forms an electron-bifurcating complex with the heterodisulfide reductase (Hdr A-C) (Takami *et al.*, 2012; Nobu *et al.*, 2015a). Polysulfide could be reduced to generate H_2_S during this process (Hedderich *et al.*, 1999; Kirkegaard *et al.*, 2016). The cytoplasmic bidirectional hydrogenases (HoxEFUHY and HymAB) catalyze electron transfer between H^+^ and electron carriers, which may function as electron valves balancing reductant in the cell (Eckert *et al.*, 2012; Cassier-Chauvat *et al.*, 2014; Fritsch *et al.*, 2013). There is a membrane-integral pyrophosphatase (HppA) which can also translocate H^+^ or Na^+^ to generate PMF using the energy produced from hydrolysis of pyrophosphate (PPi) (Luoto *et al.*, 2013). Acetothermia encodes an anti-oxidation system to respond to oxidative stress (from O_2_, superoxide O_2_^●-^, and peroxide H_2_O_2_). This system includes superoxide reductase (SorA), rubrerythrin-2 (Rbr), NAD(P)H-rubredoxin oxidoreductase (NROR) using rubredoxin (Rub) as the electron carrier; and thioredoxin reductase (Trx), hydroperoxide reductase (Prx) using thioredoxin (Trx) (Wood *et al.*, 2003; Erlendsson *et al.*, 2003). Substrates for Trx may include oxidized proteins and some transcription factors as well; and peroxiredoxin (Prx) could probably also use glutaredoxin (GSH) as electron carriers (Wood *et al.*, 2003). Unknown electron acceptor in CO oxidation and metabolism of disulfide (RS-SR′) regeneration from dithiol (RSH + R′SH) are indicated by question marks, which require further investigation to be elucidated.

# **Supplementary tables**

## Table S1: Operational and performance conditions of the investigated digesters, with relative abundance of Acetothermia. Median values of each parameter in year 2015 are present. Raw data were directly collected from digester operators at wastewater treatment plants.

| **WWTPs** | **Reactor type** | **Process** | **Main Substrate** | **Number of digesters investigated** | **Volume** | **Temperature** | **pH** | **PO_4_-P** | **NH_4_^+^** | **VFA** | **Alkalinity** |
| --- | --- | --- | --- | --- | --- | --- | --- | --- | --- | --- | --- |
|  |  |  |  |  | **m^3^** | **°C** |  | **mg/L** | **mg/L** | **mmol-acetate/L** | **mmol-HCO_3_^-^/L** |
| Avedøre | 1 stage, CSTR | Mesophilic | Sewage sludge PS+BS^1^ | 2 | 6000 | 35.6 | 7.1 | NA | 532 | 2.2 | 48.4 |
| AalborgØst | 1 stage, CSTR | Mesophilic | Sewage sludge BS | 1 | 1500 | 39.0 | 7.8 | NA | NA | NA | NA |
| Billund | 1 stage, CSTR | Mesophilic | Sewage sludge + food waste | 1 | NA^3^ | 40.0 | NA | NA | NA | NA | NA |
| Damhusåen | 1 stage, CSTR | Mesophilic | Sewage sludge PS+BS | 4 | 1900 | 35.4 | 7.1 | 119.9 | NA | 5.0 | 67.0 |
| EjbyMølle | 1 stage, CSTR | Mesophilic | Sewage sludge PS+BS | 1 | 2800 | 39.5 | 7.9 | NA | 1273 | 5.0 | 83.9 |
| **Esbjerg** | **2 stages, CSTR** | **Mesophilic** | **Sewage sludge PS+BS for Stage 1 (RT1 and 2); hydrolyzed, digested sewage sludge for Stage 2 (RT3 and 4)** | **4** | 2200-2500 | **40.3** | **7.8** | **52.5** | **1189** | **5.4** | **73.4** |
| Fornæs | 1 stage, CSTR | Mesophilic | Sewage sludge BS | 1 | 1400 | 34.5 | 7.5 | NA | 930 | 2.1 | 18.6 |
| Hjørring | 1 stage, CSTR | Mesophilic | Sewage sludge PS+BS | 1 | 1600 | 37.0 | 7.2 | NA | NA | NA | NA |
| Mariagerfjord | 1 stage, CSTR | Mesophilic | Sewage sludge BS | 1 | 2000 | 38.7 | 7.4 | 406.0 | 1568 | 6.4 | 107.5 |
| Mølleåværket | 1 stage, CSTR | Mesophilic | Sewage sludge PS+BS | 1 | 1000 | 37.0 | 7.3 | NA | NA | 0.4 | 79.0 |
| **Randers** | **1 stage, CSTR** | **Mesophilic** | **Sewage sludge PS + BS** | **2** | 2400 | **38.0** | **7.4** | **58.8** | **770** | **1.7** | **69.4** |
| Slagelse | 1 stage, CSTR | Mesophilic | Sewage sludge PS + BS | 2 | 1300-1700 | 39.0 | 7.2 | 72.7 | 741 | 2.6 | 77.0 |
| Søholt | 1 stage, CSTR | Mesophilic | Sewage sludge BS | 1 | 2340 | 40.2 | 7.6 | 134.0 | 1612 | 2.5 | 114.0 |
| Viborg | 1 stage, CSTR | Mesophilic | Sewage sludge PS+BS | 2 | 1600 | 36.6 | 7.1 | 94.6 | 489 | 2.1 | 44.0 |
| Åby | 1 stage, CSTR | Mesophilic | Sewage sludge BS | 1 | 1300 | 49.1 | 7.3 | 345.5 | 1020 | 4.7 | 64.0 |
| Fredericia | 1 stage, CSTR | Mesophilic-THP^2^ | Hydrolized sewage sludge BS | 2 | 2000 | 38.8 | 7.6 | NA | 2900 | 10.0 | 205.8 |
| Næstved | 1 stage, CSTR | Mesophilic-THP | Hydrolized sewage sludge BS | 1 | 1600 | 40.0 | 7.6 | 480.0 | 1170 | NA | NA |
| AalborgVest | 1 stage, CSTR | Thermophilic | Sewage sludge BS | 2 | 2500 | 55.0 | 7.8 | NA | 2800 | NA | NA |
| Billund | 1 stage, CSTR | Thermophilic-THP | Hydrolized sewage sludge + industrial waste | 1 | NA | 55.0 | NA | NA | NA | NA | NA |

(continued)

| **WWTPs** | **TS of Digester sludge** | **VS of Digester sludge** | **Influent TS** | **Influent VS** | **Retention time** | **OLR-TS** | **OLR-VS** | **TS reduction** | **VS reduction** | **CH_4_ content** | **CH_4_ production rate** | **Acetothermia read abundance^5^** |
| --- | --- | --- | --- | --- | --- | --- | --- | --- | --- | --- | --- | --- |
|  | **%** | **% of TS** | **%** | **% of TS** | **day** | **kg-TS/(m^3^ ·day)** | **kg-VS/(m^3^ ·day)** | **%** | **%** | **%** | **Nm^3^-CH_4_/(kg-VS day)** | **%** |
| Avedøre | 1.6 | 60.9 | NA | NA | 26.9 | 1.3 | 1.0 | NA | NA | NA | 0.26 | 0.0 |
| AalborgØst | 2.9 | NA | NA | NA | NA | NA | NA | NA | NA | NA | 0.40 | 0.0 |
| Billund | NA | NA | NA | NA | NA | NA | NA | NA | NA | NA | NA | 0.0 |
| Damhusåen | 3.3 | 56.4 | NA | NA | NA | NA | NA | NA | NA | NA | NA | 0.0 |
| EjbyMølle | 1.8 | 62.8 | NA | NA | 32.0 | NA | NA | NA | NA | 63.6 | 0.47 | 0.0 |
| **Esbjerg^4^** | **1.8** | **61.5** | **4.1** | **78.5** | **27.6** | **1.9** | **1.4** | **52.4** | **18.3** | **63.6** | **0.49** | **4.1** |
| Fornæs | 4.0 | 66.5 | 4.9 | 73.7 | NA | NA | NA | NA | NA | NA | NA | 0.0 |
| Hjørring | 3.0 | NA | NA | NA | 37.3 | 1.5 | 1.1 | NA | NA | NA | 0.36 | 0.0 |
| Mariagerfjord | 4.1 | 64.6 | 8.0 | NA | 42.9 | 2.1 | 1.5 | 45.4 | NA | NA | 0.27 | 0.0 |
| Mølleåværket | 3.8 | 57.0 | 5.9 | 73.8 | NA | NA | NA | NA | NA | NA | NA | 0.0 |
| **Randers** | **3.5** | **58.0** | **5.6** | **82.5** | **34.5** | **1.6** | **1.2** | **46.4** | **29.9** | **62.5** | **0.23** | **7.7** |
| Slagelse | 2.7 | 58.0 | NA | NA | 60.1 | 1.0 | 0.8 | NA | NA | NA | 0.35 | **0.1** |
| Søholt | 5.1 | 63.0 | 7.1 | NA | 28.7 | 2.3 | 1.7 | 27.3 | NA | 63.8 | 0.25 | 0.0 |
| Viborg | 2.0 | 59.0 | 2.7 | NA | 24.6 | 1.2 | 0.9 | 32.0 | NA | 61.8 | 0.38 | 0.0 |
| Åby | 3.7 | 63.5 | 4.6 | 74.0 | 16.2 | 3.0 | 2.2 | 19.4 | 14.9 | 53.4 | 0.16 | 0.0 |
| Fredericia | 6.4 | 53.2 | 9.0 | 67.0 | 35.0 | 3.0 | 2.3 | 26.4 | 20.4 | NA | 0.21 | 0.0 |
| Næstved | 3.7 | NA | 5.5 | NA | 16.0 | 3.4 | 2.6 | 33.1 | NA | 62.7 | 0.31 | 0.0 |
| AalborgVest | NA | NA | NA | NA | NA | NA | NA | NA | NA | NA | NA | 0.0 |
| Billund | NA | NA | NA | NA | NA | NA | NA | NA | NA | NA | NA | 0.0 |

^1^ PS = Primary Sludge; BS = Biological Sludge.

^2^ THP = Thermal Hydrolysis Process, used for pretreating the sludge before it is fed into the digesters.

^3^ NA = Not available.

^4^ Rows highlighted in red show the plants in which Acetothermia bacteria were detected.

^5^ Acetothermia read abundance was calculated as an average percentage of amplicons assigned to Acetothermia for each WWTP in 2015, derived from a survey into the microbial community composition of digesters with amplicon sequencing method.

## Table S2: Characteristics of the metagenomic data sets.

| **Sample and sequencing information** | **Sample name** | **Sampling Date** | **WWTP, Digester** | **Kit for Library preparation** | **Sequencing platform** |
| --- | --- | --- | --- | --- | --- |
|  | S2 | 20160512 | Randers, RT2 | Nextera DNA | Hiseq |
|  | S3 | 20160323 | Randers, RT2 | Nextera DNA | Hiseq |
|  | S4 | 20160113 | Randers, RT2 | Nextera DNA | Miseq |
|  | S5 | 20160321 | Randers, RT2 | Nextera DNA | Miseq |
|  | S6 | 20160323 | Randers, RT2 | SQK-LSK108, Ligation Sequencing Kit 1D | Nanopore |
| **Characteristic of sequencing datasets (after trimming)** | **Sample name** | **Number of reads** | **Average read length** | **Size of metagenome (Mbp)** | **Estimated coverage^1^** |
|  | S2 | 27,571,325 | 223 | 6,137 | 24.5 |
|  | S3 | 38,331,609 | 208 | 7,988 | 32.0 |
|  | S4 | 10,128,405 | 213 | 2,160 | 8.6 |
|  | S5 | 12,515,654 | 211 | 2,645 | 10.6 |
|  | S6 | 674,338 | 3,412 | 2,301 | 9.2 |
| **Characteristic of genome binning** | **Sample name** | **Metagenome size (Mbp)** | **Total length of sequences mapped to Acetothermia genome bin**^2^ **(Mbp)** | **Coverage** | **Acetothermia abundance (%, based on coverage)** |
|  | S2 | 3,650 | 82 | 62 | 2.3 |
|  | S3 | 4,991 | 297 | 224 | 6.0 |
|  | S4 | 1,476 | 83 | 64 | 5.6 |
|  | S5 | 1,715 | 114 | 87 | 6.6 |
|  | S6 |  |  |  |  |

## ^1^ Coverage was roughly estimated assuming genome size is 5 Mbp and abundance 2%.

## ^2^ Calculated by: total length of Acetothermia genome bin × coverage value (in each metagenome).

## Table S3: Properties of the Acetothermia sp. Ran1 genome.

| **Genome bin identifier** | **Ran1** |
| --- | --- |
| Source digester | Randers WWTP-RT2 |
| Closest environmental 16S clone | Er-MLAYS-81 (EU542484)^1^ |
| Total length (bp) | 1,324,338 |
| GC (%) | 68.2 |
| Number of tRNA genes | 46 |
| rRNA genes found in genome | 5S, 16S, 23S; one copy for each |
| Number of CDS | 1327 |
| Number of CRISPR array | 0 |
| Coding density (%) | 93.4 |
| Average CDS length (bp) | 947 |
| Average intergenic length (bp) | 87 |

^1^ Calculated with nucleotide blast against nucleotide collection (nr/nt) database in NCBI.

## Table S4: FISH probes designed to target bacteria related to the Ran1 genus-level lineage.

| **Probe^1^** | ***E. coli* pos.** | **Sequence (5’-3’)** | **[FA]%^2^** |
| --- | --- | --- | --- |
| **OP1-702** | **702-722** | **TCG GGR TAC CGA CCG GTA TCT** | **40** |
| OP1-702_H1 (helper probe) | 678-701 | ACG CAT TYC ACC GCT ACT CCG GTC |  |
| OP1-702_H2 (helper probe)^3^ | 723-744 | ACC CAG AAG GTT GCC TTC GCC A |  |
| **OP1-838** | **838-857** | **GGC ACC AAG CGA CTG CTC G** | **40** |
| OP1-838_H1 (helper probe)^3^ | 818-837 | CCC GAC ACC TAG CCC ACA TC |  |
| OP1-838_H2 (helper probe) | 858-881 | CGG TGG GCT TAA CGC GTT AGC TGC |  |

^1^ Both probes cover all near full-length sequences of the proposed genus (**Figure S4**) and do not hit any non-target sequences in the SILVA SSURef NR 99 database (release 128). There were no non-target hits for either probe.

^2^ Optimal hybridization formamide concentration % [v/v].

^3^ Addition of these helper probes is not necessary to improve fluorescence.

# **Supplementary Data**

## Supplementary Data Set1: Metadata of amplicon sequencing datasets (separate file).

## Supplementary Data Set2: List of curated annotations for Acetothermia sp. Ran1 genome (separate file).

## Supplementary Data Set3: Titles and reference IDs for 16S rRNA gene sequences used in Figure S1, which are affiliated with Acetothermia within the SILVA database (separate file).

# **Supplementary references:**

Adams MWW, Holden JF, Menon AL, Schut GJ, Grunden AM, Hou C, *et al.* (2001). Key role for sulfur in peptide metabolism and in regulation of three hydrogenases in the hyperthermophilic archaeon *Pyrococcus furiosus*. *J Bacteriol* **183**: 716–724.

Albertsen M, Hugenholtz P, Skarshewski A, Nielsen KL, Tyson GW, Nielsen PH. (2013). Genome sequences of rare, uncultured bacteria obtained by differential coverage binning of multiple metagenomes. *Nat Biotechnol* **31**: 533–538.

Albertsen M, Karst SM, Ziegler AS, Kirkegaard RH, Nielsen PH. (2015). Back to basics - the influence of DNA extraction and primer choice on phylogenetic analysis of activated sludge communities. *PLoS One* **10**: e0132783.

Bolger AM, Lohse M, Usadel B. (2014). Trimmomatic: a flexible trimmer for Illumina sequence data. *Bioinformatics* **30**: 2114–20.

Bridger SL, Andrew Lancaster W, Poole FL, Schut GJ, Adams MWW. (2012). Genome sequencing of a genetically tractable *Pyrococcus furiosus* strain reveals a highly dynamic genome. *J Bacteriol* **194**: 4097–4106.

Brines LM, Kovacs JA. (2007). Understanding the mechanism of superoxide reductase promoted reduction of superoxide. *Eur J Inorg Chem* **2007**: 29–38.

Buckel W, Thauer RK. (2013). Energy conservation via electron bifurcating ferredoxin reduction and proton/Na^+^ translocating ferredoxin oxidation. *Biochim Biophys Acta - Bioenerg* **1827**: 94–113.

Caporaso JG, Lauber CL, Walters WA, Berg-lyons D, Huntley J, Fierer N, *et al.* (2012). Ultra-high-throughput microbial community analysis on the Illumina HiSeq and MiSeq platforms. *ISME J* **6**: 1621–1624.

Caporaso JG, Lauber CL, Walters WA, Berg-Lyons D, Lozupone CA, Turnbaugh PJ, *et al.* (2011). Global patterns of 16S rRNA diversity at a depth of millions of sequences per sample. *Proc Natl Acad Sci* **108**: 4516–4522.

Carrondo MA, Andrews S, Arosio P, Levi S, Banyard S, Stammers D, *et al.* (2003). Ferritins, iron uptake and storage from the bacterioferritin viewpoint. *EMBO J* **22**: 1959–68.

Cassier-Chauvat C, Veaudor T, Chauvat F. (2014). Advances in the function and regulation of hydrogenase in the cyanobacterium *Synechocystis* PCC6803. *Int J Mol Sci* **15**: 19938–19951.

Chaganti SR, Lalman JA, Heath DD. (2012). 16S rRNA gene based analysis of the microbial diversity and hydrogen production in three mixed anaerobic cultures. *Int J Hydrogen Energy* **37**: 9002–9017.

Chen IA, Markowitz VM, Chu K, Palaniappan K, Szeto E, Pillay M, *et al.* (2017). IMG/M : integrated genome and metagenome comparative data analysis system. *Nucleic Acids Res* **45**: 507–516.

Consortium J, Microbiome H, Data P, Working G. (2012). Evaluation of 16S rDNA-based community profiling for human microbiome research. *PLoS One* **7**. e-pub ahead of print, doi: 10.1371/journal.pone.0039315.

Copeland A, Zeytun A, Yassawong M, Nolan M, Lucas S, Hammon N, *et al.* (2012). Complete genome sequence of the orange-red pigmented, radioresistant *Deinococcus proteolyticus* type strain (MRPT). *Stand Genomic Sci* **6**: 240–50.

Daims H, Stoecker K, Wagner M. (2005). Fluorescence in situ hybridization for the detection of prokaryotes. *Mol Microb Ecol* **213**: 239.

Eckert C, Boehm M, Carrieri D, Yu J, Dubini A, Nixon PJ. (2012). Genetic analysis of the Hox hydrogenase in the cyanobacterium *Synechocystis* sp. PCC 6803 reveals subunit roles in association, assembly, maturation, and function. *J Biol Chem* **287**: 43502–43515.

Edgar RC. (2010). Search and clustering orders of magnitude faster than BLAST. *Bioinformatics* **26**: 2460–2461.

Erlendsson LS, Acheson RM, Hederstedt L, Le Brun NE. (2003). Bacillus subtilis ResA is a thiol-disulfide oxidoreductase involved in cytochrome c synthesis. *J Biol Chem* **278**: 17852–17858.

Finn RD, Coggill P, Eberhardt RY, Eddy SR, Mistry J, Mitchell AL, *et al.* (2016). The Pfam protein families database: towards a more sustainable future. *Nucleic Acids Res* **44**: D279–D285.

Fritsch J, Lenz O, Friedrich B. (2013). Structure, function and biosynthesis of O₂-tolerant hydrogenases. *Nat Rev Microbiol* **11**: 106–14.

Fukui T, Atomi H, Kanai T, Matsumi R, Fujiwara S, Imanaka T. (2005). Complete genome sequence of the hyperthermophilic archaeon *Thermococcus kodakaraensis* KOD1 and comparison with *Pyrococcus* genomes. *Genome Res* **15**: 352–363.

Goux X, Calusinska M, Fossépré M, Benizri E, Delfosse P. (2016). Start-up phase of an anaerobic full-scale farm reactor - appearance of mesophilic anaerobic conditions and establishment of the methanogenic microbial community. *Bioresour Technol* **212**: 217–226.

Goux X, Calusinska M, Lemaigre S, Marynowska M, Klocke M, Udelhoven T, *et al.* (2015). Microbial community dynamics in replicate anaerobic digesters exposed sequentially to increasing organic loading rate, acidosis, and process recovery. *Biotechnol Biofuels* **8**: 122.

Hedderich R, Klimmek O, Kro A, Dirmeier R, Keller M, Stetter KO. (1999). Anaerobic respiration with elemental sulfur and with disulfides. *FEMS Microbiol Rev* **22**: 353–381.

Herlemann DPR, Labrenz M, Ju K, Bertilsson S, Waniek JJ, Andersson AF. (2011). Transitions in bacterial communities along the 2000 km salinity gradient of the Baltic Sea. *ISME J* **5**: 1571–1579.

Hu P, Tom L, Singh A, Thomas B, Baker B, Piceno Y, *et al.* (2016). Genome-resolved metagenomic analysis reveals roles for candidate phyla and other microbial community members in biogeochemical transformations in oil reservoirs. *MBio* **7**: 1–12.

Hyun SL, Sung GK, Seung SB, Jae KL, Cho Y, Yun JK, *et al.* (2008). The complete genome sequence of *Thermococcus onnurineus* NA1 reveals a mixed heterotrophic and carboxydotrophic metabolism. *J Bacteriol* **190**: 7491–7499.

Kirkegaard RH, Dueholm MS, McIlroy SJ, Nierychlo M, Karst SM, Albertsen M, *et al.* (2016). Genomic insights into members of the candidate phylum Hyd24-12 common in mesophilic anaerobic digesters. *ISME J* **10**: 1–13.

Klindworth A, Pruesse E, Schweer T, Peplies J, Quast C, Horn M, *et al.* (2013). Evaluation of general 16S ribosomal RNA gene PCR primers for classical and next-generation sequencing-based diversity studies. *Nucleic Acids Res* **41**: 1–11.

Kwon S, Kim TS, Yu GH, Jung JH, Park HD. (2010). Bacterial community composition and diversity of a full-scale integrated fixed-film activated sludge system as investigated by pyrosequencing. *J Microbiol Biotechnol* **20**: 1717–1723.

Luoto HH, Baykov A a, Lahti R, Malinen AM. (2013). Membrane-integral pyrophosphatase subfamily capable of translocating both Na^+^ and H^+^. *Proc Natl Acad Sci U S A* **110**: 1255–60.

Magoč T, Salzberg SL. (2011). FLASH: fast length adjustment of short reads to improve genome assemblies. *Bioinformatics* **27**: 2957–2963.

McIlroy SJ, Kirkegaard RH, McIlroy B, Nierychlo M, Kristensen JM, Karst SM, *et al.* (2017). MiDAS 2.0: an ecosystem-specific taxonomy and online database for the organisms of wastewater treatment systems expanded for anaerobic digester groups. *Database* **2017**: 1–9.

Nobu MK, Narihiro, T., Kuroda K, Mei R, Liu WT. (2016). Chasing the elusive Euryarchaeota class WSA2: genomes reveal a uniquely fastidious methyl-reducing methanogen. *ISME J* **2**: 1–10.

Nobu MK, Narihiro T, Hideyuki T, Qiu YL, Sekiguchi Y, Woyke T, *et al.* (2015a). The genome of *Syntrophorhabdus aromaticivorans* strain UI provides new insights for syntrophic aromatic compound metabolism and electron flow. *Environ Microbiol* **17**: 4861–4872.

Nobu MK, Narihiro T, Rinke C, Kamagata Y, Tringe SG, Woyke T, *et al.* (2015b). Microbial dark matter ecogenomics reveals complex synergistic networks in a methanogenic bioreactor. *ISME J* **9**: 1710–1722.

Perkins SD, Scalfone NB, Angenent LT. (2011). Comparative 16S rRNA gene surveys of granular sludge from three upflow anaerobic bioreactors treating purified terephthalic acid (PTA) wastewater. *Water Sci Technol* **64**: 1406–1412.

Quast C, Pruesse E, Yilmaz P, Gerken J, Schweer T, Yarza P, *et al.* (2013). The SILVA ribosomal RNA gene database project: improved data processing and web-based tools. *Nucleic Acids Res* **41**: 590–596.

R Core Team. (2016). R: a language and environment for statistical computing. R Foundation for Statistical Computing, Vienna: Vienna, Austria.

Rosenberg E, DeLong EF, Lory S, Stackebrandt E, Thompson F, Lory S, *et al.* (2013). The prokaryotes: prokaryotic physiology and biochemistry. 4th ed. Springer-Verlag Berlin Heidelberg.

Takami H, Noguchi H, Takaki Y, Uchiyama I, Toyoda A, Nishi S, *et al.* (2012). A deeply branching thermophilic bacterium with an ancient Acetyl-CoA pathway dominates a subsurface ecosystem. *PLoS One* **7**: e30559.

Wallner G, Amann R, Beisker W. (1993). Optimizing fluorescent *in situ* hybridization with rRNA-targeted oligonucleotide probes for flow cytometric identification of microorganisms. *Cytometry* **14**: 136–143.

Wang Q, Garrity GM, Tiedje JM, Cole JR. (2007). Naïve Bayesian classifier for rapid assignment of rRNA sequences into the new bacterial taxonomy. *Appl Environ Microbiol* **73**: 5261–5267.

Wickham H. (2009). ggplot2: elegant graphics for data analysis. Springer-Verlag New York: New York.

Wood ZA, Schröder E, Harris JR, Poole LB. (2003). Structure, mechanism and regulation of peroxiredoxins. *Trends Biochem Sci* **28**: 32–40.
